# Research Progress of Persistent Luminescence Nanoparticles in Biological Detection Imaging and Medical Treatment

**DOI:** 10.3390/ma18173937

**Published:** 2025-08-22

**Authors:** Kunqiang Deng, Kunfeng Chen, Sai Huang, Jinkai Li, Zongming Liu

**Affiliations:** 1 School of Material Science and Engineering, University of Jinan, Jinan 250022, China; 2 State Key Laboratory of Crystal Materials, Institute of Novel Semiconductors, Shandong University, Jinan 250100, China

**Keywords:** persistent luminescent nanoparticles, biological detection, bioimaging, medical treatment

## Abstract

Persistent luminescence nanoparticles (PLNPs) represent a unique class of optical materials. They possess the ability to absorb and store energy from external excitation sources and emit light persistently once excitation terminates. Because of this distinctive property, PLNPs have attracted considerable attention in various areas. Especially in recent years, PLNPs have revealed marked benefits and extensive application potential in fields such as biological detection, imaging, targeted delivery, as well as integrated diagnosis and treatment. Not only do they potently attenuate autofluorescence interference arising from biological tissues, but they also demonstrate superior signal-to-noise ratio and sensitivity in in vivo imaging scenarios. Therefore, regarding the current research, this paper firstly introduces the classification, synthesis methods, and luminescence mechanism of the materials. Subsequently, the research progress of PLNPs in biological detection and imaging and medical treatment in recent years is reviewed. The challenges faced by materials in biomedical applications and the outlook of future development trends are further discussed, which delivers an innovative thought pattern for developing and designing new PLNPs to cater to more practical requirements.

## 1. Introduction

Biomedical research is inherently intertwined with human health, where technological advancements in bio-detection and imaging stand as pivotal pillars of modern precision medicine [1,2,3]. Traditional clinical imaging methods include computed tomography (CT), positron emission tomography (PET), and magnetic resonance imaging (MRI). CT boasts favorable resolution but is constrained by radiation risks and high costs; MRI offers distinct safety advantages yet suffers from low sensitivity and motion-induced artifacts; PET provides higher sensitivity and quantitative detection capabilities compared to the other two, though it is hindered by high costs and limited instrumentation accessibility [4,5,6]. In contrast, optical imaging based on fluorescence principles has garnered extensive attention in biosensing, cancer diagnosis, and drug development owing to its advantages of high sensitivity, rapid detection, non-invasiveness, and operational simplicity [7,8].

Optical imaging techniques mainly use optical signal dyes conjugated with biorecognition receptors to construct fluorescent probes that are able to bind specifically to the target, enabling the labeling, monitoring, and evaluation of cells or tissues associated with living subjects [9,10]. It has been widely applied in biosensing, cancer diagnosis, and therapy as well as drug development and delivery. However, with the deepening of research, the limitations of nanoprobes composed of conventional fluorescent agents such as organic dyes (small-molecule organic compounds), semiconducting polymers (organic macromolecules), and upconverted nanoparticles (inorganic nanomaterials) are gradually manifested. The majority of these nanoprobes typically suffer from issues like photobleaching, poor signal-to-noise ratio (SNR), and weak fluorescence intensity, thereby posing substantial challenges for their practical applications [11]. Moreover, the luminescence lifetime of traditional fluorescent materials is short, generally from nanoseconds to microseconds, which makes them difficult to distinguish from the background fluorescence of biological tissues in the detection process of complex biological systems, and it is easy to be submerged by background noise, which increases the difficulty of signal processing and analysis [12,13,14]. Therefore, the development of a high-performance luminescent material for biomedical applications to achieve efficient, accurate, and low side effects of biological detection, imaging, and medical treatment has always been the goal pursued by researchers.

Persistent luminescent nanoparticles (PLNPs), also referred to as afterglow materials, are a unique class of photoluminescent materials. They can absorb and store energy from excitation sources such as ultraviolet light, visible light, near-infrared radiation, and X-rays [15,16,17]. Following the cessation of excitation, the stored energy is gradually emitted in the form of photons, with the afterglow signal persisting for minutes, hours, or even days [16,18,19]. Due to their distinctive luminescent properties, PLNPs find wide applications in safety signage, temperature sensing, and optical information storage. Notably, with the advancement of nanotechnology, PLNPs have evolved from bulk materials (long persistent luminescent materials, LPLMs) to ultrasmall nanoscale particles, enabling precise surface functionalization to enhance biocompatibility, stability, and targeting efficiency. These improvements have expanded their application potential in biomedical fields, including biological detection, targeted delivery, immunotherapy, and integrated diagnosis and therapy [20,21,22,23]. Among them, in the process of biological detection and imaging, PLNPs can efficiently evade the disturbances from light damage, photobleaching, background fluorescence, and biological tissue autofluorescence; improve the sensitivity of biological detection and the SNR of imaging so as to achieve clearer and more accurate imaging and detection; and provide strong support for the early diagnosis of diseases [24,25]. In addition, in medical treatment, PLNPs can not only be used as carriers for drug delivery but also as photosensitizers or photothermal conversion agents for photodynamic therapy and photothermal therapy so as to achieve precise management of tumor cells, improve drug treatment outcomes, and minimize harm to normal tissues [26,27,28]. However, biomedical applications impose stringent requirements on the size, monodispersity, uniformity, and afterglow properties of PLNPs. Accordingly, the development of controllable synthesis strategies for PLNPs is of paramount importance. This is because the synthetic approach directly dictates critical material attributes, including microstructural features, afterglow behavior, fluorescence quantum yield, and defect distribution. These characteristics, in turn, serve as fundamental prerequisites for the successful implementation of PLNPs in biomedical scenarios. Consequently, sustained efforts to explore innovative synthesis routes and optimize existing protocols remain indispensable. Such endeavors are crucial for enhancing the overall performance of PLNPs, thereby expanding their potential in advanced biomedical applications.

**Figure 1:**
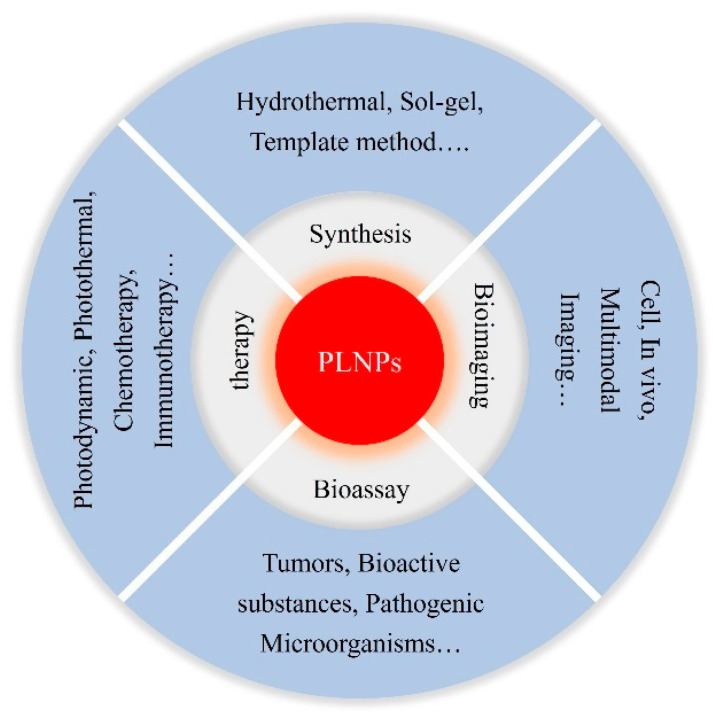
Summary of biomedical applications of PLNPs.

Given the distinctive luminescent properties and significant biomedical utility of PLNPs, a number of review articles have been published on this topic in relevant literature. For instance, in 2023, Yang et al. focused their discussion on the application of non-UV-excited PLNPs in bioimaging [17], while in 2024, Chen et al. reviewed the use of NIR PLNPs in cancer therapy [25]. Building on existing research findings, this paper aims to provide a comprehensive review of the latest advancements in the biomedical applications of inorganic PLNPs, covering multiple aspects of bio-detection, imaging, and medical therapy. In this review, we systematically summarize the classification, synthesis methods, and luminescence mechanisms of PLNPs and comprehensively discuss their cutting-edge applications in biological detection, biological imaging, and medical treatment (Figure 1). We also conduct an in-depth analysis of current technical bottlenecks and provide an outlook on future developments. It is our hope that this review will further stimulate research vitality and innovation potential in the field of PLNPs for biomedical applications and offer new insights for advancing related technological innovations, upgrading medical testing methods, and optimizing therapeutic strategies.

## 2. Overview of Long Persistent Luminescent Materials

**Figure 2:**
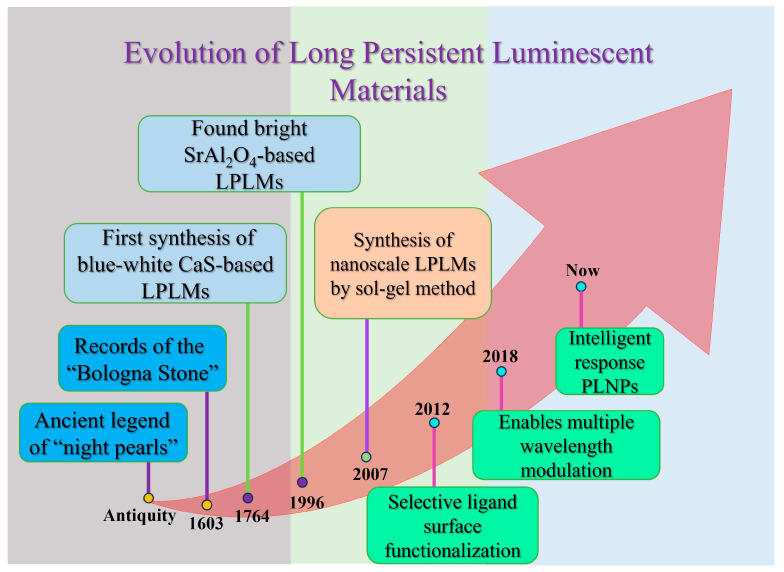
Development of long persistent luminescent materials.

LPLMs have a long history, and their research and application can be traced back to ancient times. Figure 2 outlines their development. It is understood that at that time, it was found that many natural ores themselves could absorb sunlight and emit light in the dark, with long afterglow luminescence characteristics, so they were made into a series of exquisite items such as luminous cups and night pearls. During the Song Dynasty of China, an example of drawing a cattle painting with oysters made of long afterglow pigments was recorded [16]. The cattle in the painting were still clearly visible at night. The study of such materials in the West first occurred in 1603. When an Italian shoemaker roasted the local ore for gold smelting, he obtained some materials that glowed red in the night. Later analysis showed that the ore contained barium sulfate. After reduction roasting, it became a barium sulfide LPLM, so this ore is also called Bologna stone [29,30]. In 1764, British researchers prepared a blue-white luminescent material through firing a mixture of oyster shells and sulfur, which was identified as a calcium sulfide LPLM. In 1866, Sidot from France completed the preparation of ZnS: Cu, initiating systematic research on long-persistent luminescent materials [16,29,30,31]. Subsequently, ZnS: Cu became an early industrial persistent phosphor, dominating the market with diverse applications. In addition, researchers also extensively studied similar alkaline earth sulfides (CaS, SrS, BaS) doped with rare earth ions or transition metal ions (Cr^3+^, Dy^3+^, Eu^2+^). A pivotal advancement occurred in 1996, when Matsuzawa and colleagues reported a novel metal oxide LPLM. It was composed of Eu and Dy co-doped SrAl_2_SO_4_, which could exhibit bright green illumination with an afterglow life of 2000 min [32]. This discovery marked a new stage of LPLM development. In 2007, Chermont prepared Ca_0.2_Zn_0.9_Mg_0.9_Si_2_O_6_: Eu^2+^, Dy^3+^, Mn^2+^ nanoparticles via the sol-gel method, marking the shift of LPLM research from bulk or powder formation to the nanoscale [33]. Besides, they also demonstrated proof-of-concept real-time, excitation-free bioimaging in mice using these PLNPs. In 2012, Maldiney synthesized PLNPs with PEG coating and biotin surface modification. Through the highly specific interaction between biotin and avidin, these PLNPs were successfully applied to detect glioma cells for a definitive therapy [34]. Nowadays, PLNPs are developing towards diversification and smart response, showing better luminescence performance. This will usher in a new era of PLNP applications.

### 2.1. Classification of LPLMs

There are many types of LPLMs. According to the composition and structural characteristics, they are primarily classified into three categories: inorganic LPLMs, organic LPLMs, and organic-inorganic hybrid LPLMs.

#### 2.1.1. Inorganic LPLMs

Inorganic LPLMs were the first to be studied, and their applications are also the most widespread. These materials are usually based on inorganic compounds doped with specific rare earth ions (such as Eu^2+^, Dy^3+^) or other activators (such as Mn^2+^, Cr^3+^) as luminescent centers to achieve afterglow luminescence performance [35,36]. Common matrixes for inorganic LPLMs include aluminates, silicates, gallate, and sulfide. Different matrix materials give them unique structural and optical properties. Table 1 summarizes the afterglow performance exhibited by various matrixes, from which it can be seen that the excitation and emission spectra of inorganic LPLMs have been covered to the ultraviolet, visible, and near-infrared ranges, and they are able to achieve a diversity of luminescent colors [18]. Furthermore, the luminescence intensity, afterglow lifetime, and wavelength of inorganic LPLMs can be balanced between particle size and morphology by changing the synthesis method and adjusting the synthesis conditions so as to obtain LPLMs with high luminescence performance and a controllable shape and size. However, inorganic LPLMs still have some limitations. The preparation processes of some materials are relatively complex, which not only increases production costs but may also affect their performance to a certain extent [37,38]. Therefore, we need to optimize the preparation process of materials and explore new synthesis methods. Additionally, some inorganic LPLMs exhibit poor biocompatibility and contain heavy metal elements such as lead and cadmium, which may pose potential toxicity to organisms. Thus, researchers need to actively develop new lead-free and non-toxic inorganic LPLMs. For the purpose of further enlarging the uses of LPLMs in biomedicine, researchers can also improve the optical properties and stability of the materials by means of doping, nanosizing, surface coating, and surface functionalization to enhance biocompatibility and environmental friendliness so that the materials can better meet practical needs.

#### 2.1.2. Organic LPLMs

Organic LPLMs are a new class of LPLMs developed in recent years, which have attracted much more attention from researchers due to their flexible designability, tunable emission wavelengths, easy preparation, low cost, and good biocompatibility [56,57,58]. These materials mainly include organic small molecules, organic macromolecules, and polymers. In contrast to conventional inorganic materials, the light-emission characteristics of organic LPLMs are readily tunable through the strategic manipulation of π-conjugated constituents moiety aggregation or via the rational design of the molecular structures. Fundamental mechanisms underpinning the afterglow in these materials encompass chemical defects and energy gaps. Taking small organic molecules as an example, these materials are generally composed of aromatic compounds or heterocyclic compounds (such as carbazole and benzophenone derivatives). These molecular derivatives typically possess rigid conjugated systems and rely on intramolecular electron transitions and energy transfer processes. When stimulated by light, electrons undergo a transition from the base state to the excited singlet, and through an intersystem crossing (ISC) process, they convert to the excited triplet, achieving afterglow luminescence [59,60]. In 2013, Tang prepared a range of benzophenone derivatives. It was found that multiple interactions in crystalline molecules can restrict intramolecular rotations, substantially stiffen the molecular conformation, and notably reduce the nonradiative deactivation pathways of triplet excitons, thereby yielding enhanced phosphorescent emission with quantum efficiencies reaching 39.7% at room temperature [61]. In 2015, Huang et al. utilized the H-aggregation strategy to lower the triplet energy levels, stabilize the triplet excitons, and reduce the triplet radiative decay at room temperature. Meanwhile, through modulation of molecular structures, they achieved phosphorescence color transformation and ultimately obtained a series of carbazole molecules with ultra-long afterglow characteristics [62]. In 2017, Li reported a range of compounds based on carbazolyl phenyl ketone, in which different substituents were used to regulate stacking degrees, enabling ultra-long-lived phosphorescence emission [63]. In 2020, Chi et al. achieved dynamic ultra-long organic phosphorescence emission in diphenyl sulfone molecular crystals through the synergy of intramolecular and intermolecular interactions [64]. Although organic small-molecule LPLMs have a clear molecular structure, high phosphorescence efficiency, and narrow emission spectra, allowing precise regulation to achieve pure luminescent colors, their weak intermolecular interactions make triplet excitons prone to returning to the ground state via non-radiative transitions, resulting in very short afterglow lifetimes. In 2022, Yan et al. designed metal-cytosine halide hybrids through molecular engineering, realizing ultra-long afterglow and multi-mode dynamic color adjustment by modulating excitation wavelength, time evolution, and temperature to trigger proton transfer processes [65]. Organic small-molecule LPLMs have made great breakthroughs but still require continuous optimization. Currently, researchers are enhancing spin-orbit coupling effects and achieving high-performance luminescence by introducing strategies such as carbonyl groups, heteroatoms, and heavy atoms [66,67,68].

For organic macromolecular and polymeric LPLMs, their main bodies are usually composed of multiple repeating units. Their luminescent groups are introduced through covalent bonds or non-covalent interactions (van der Waals forces, hydrogen bonds, or π-π stacking) [69,70]. This complex structure provides a unique rigid environment that can effectively restrict molecular motion, stabilize triplet excitons, and reduce intermolecular energy transfer and exciton non-radiative transitions, thereby achieving afterglow emission [71]. In 2016, Tian et al. synthesized a series of polymeric phosphorescent materials through the copolymerization of acrylamide and certain phosphorescent emitters. It was found that crosslinked hydrogen bonds between polymer chains can effectively immobilize the phosphors, inhibit non-radiative decay, and protect triplet excitons, with a phosphorescence lifetime of up to 5.76 ms [72]. In 2019, Huang et al. synthesized a strategy based on traditional polyethylene derivatives, achieving ultra-long phosphorescence emission via ionic bond crosslinking and obtaining polychromatic persistent ambient-temperature phosphorescence by tuning the excitation wavelength of the polymers [73]. Although organic small molecules, organic macromolecules, and polymer LPLMs have their own characteristics in structure and performance, they can also cooperate with each other [74,75,76]. Through the polymerization reaction, small organic molecular monomers can be connected into macromolecules and then transformed into polymers. The stability of the material is often improved by moving from small organic molecules to large organic molecules or polymers. In addition, doping organic small molecules into polymers to form composite systems often results in synergistic effects as well. Such composites are more advantageous for target tracking, drug delivery, and drug release, and they also offer new options for biomedical applications.

#### 2.1.3. Organic-Inorganic Hybrid LPLMs

Beyond inorganic and organic LPLMs, researchers have leveraged the synergistic advantages of both categories to develop innovative organic-inorganic hybrid LPLMs, thereby inaugurating a new paradigm in long-lasting luminescence research. Among these hybrid systems, metal-organic framework (MOF)-based LPLMs represent the most prominent category, featuring micro/nanoscale architectures formed via coordination interactions between organic ligands and metal ions or clusters, which assemble into extended network frameworks [77]. These structural configurations not only preserve the intrinsic robust luminescent properties of inorganic components but also integrate the structural tunability characteristic of organic ligands. The afterglow emission of MOF-based LPLMs involves multiple underlying mechanisms, encompassing metal center-derived luminescence, lanthanide/actinide-mediated antenna effect-induced emission, guest molecule-triggered luminescence, ligand-originated emission, and energy transfer events between framework constituents. Within the MOF architecture, the coordination environment of metal ions facilitates exploitation of the heavy atom effect, which enhances spin-orbit coupling of triplet excitons. This process effectively suppresses non-radiative transition pathways, thereby enabling the generation of sustained afterglow emission [78]. Owing to their high porosity, excellent loading capacity, readily modifiable porous structures, and diverse compositional profiles, MOF-based LPLMs have garnered growing research attention. In 2021, Liu and colleagues reported a zinc-triazole MOF (ECUT-137) exhibiting long-lasting luminescence with a duration of up to 3 s [79]. More recently, in 2024, Yu et al. synthesized a series of lanthanide-based MOFs using lanthanide nitrates and 4-(2,5-dicarboxyphenyl) phthalate, which displayed blue afterglow with a persistence of 0.8 s [80]. Nevertheless, MOFs with long afterglow properties still face multiple challenges in practical applications. Firstly, the selection of organic ligands and metal ions applicable to the construction of long afterglow MOFs is relatively limited, and their high cost elevates the barriers to practical adoption. Secondly, some MOF materials demonstrate insufficient stability in air or aqueous environments, which may impair their long afterglow performance [81]. Therefore, further investigations into MOF-based LPLMs are thus required to drive their evolution toward enhanced convenience, higher efficiency, improved sustainability, and greater cost-effectiveness.

While inorganic, organic, and organic-inorganic hybrid LPLMs each possess distinct characteristics, the majority of research efforts remains focused on the biomedical applications of inorganic LPLMs. This preference stems from their unparalleled stability and superior long afterglow lifetimes relative to the other two categories. Through regulation and surface functionalization, it can better meet the practical application requirements of safety, long-term effect, and targeting in the biomedical field.

### 2.2. Synthesis of PLNPs

**Figure 3:**
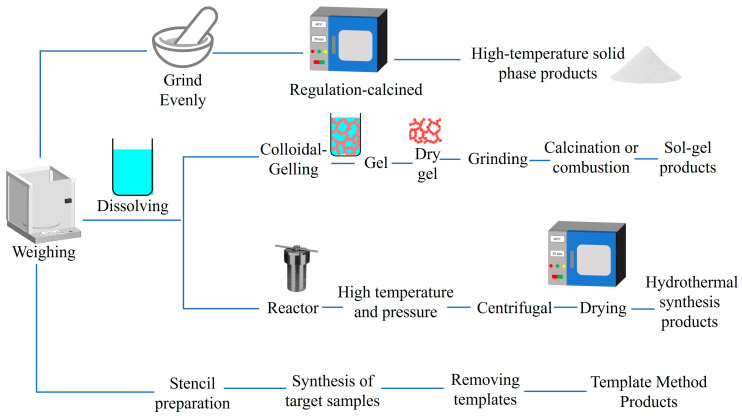
Several brief flowcharts for the preparation of PLNPs.

A good synthesis method not only has an important influence on the morphology, size, defect distribution, quantum yield, luminous efficiency, and afterglow performance of PLNPs but also further affects its application in different fields [82,83,84]. Table 2 presents a comparative analysis of the characteristics associated with different synthesis methods, whereas Figure 3 presents a concise flowchart illustrating these methodologies. Among them, the high-temperature solid-state method is the most traditional and widely used synthesis method. Through ball milling, solid-phase transformation, and high-temperature sintering of raw materials, this method can obtain certain afterglow performances of PLNPs. In 2022, Xu et al. prepared MgGa_2_O_4_ doped with different mole fractions of Bi^3+^ by the high-temperature solid phase method. After 20 s of UV excitation at 254 nm, the PLNPs exhibit a dynamic transition from green or near-infrared to blue-white light emission with good afterglow properties [85]. However, it was found by further investigation that PLNPs suffered from particle agglomeration and inhomogeneous dispersion in the micrometer range, which exposed the limitations of the solid-state method. Therefore, although the high-temperature solid-state method is simple to operate and suitable for large-scale production, it has less control over the shape and size of the product, which can easily cause agglomeration. The synthesized PLNPs have a large size, uneven distribution, and irregular morphology, which limits the application of the materials in biomedicine [24,86]. Nowadays, with continuous breakthroughs in technology, researchers have developed a variety of new synthetic methods with controllable size and morphology, including the sol-gel method, hydrothermal method, template method, and so on. These methods allow for PLNPs with more choices in preparation.

The sol-gel method is a wet chemical synthesis method, which can prepare small particles and high-purity PLNPs at a relatively low temperature. The method usually requires mixing a metal salt precursor, ligand, and crosslinking agent to form sol. Subsequently, the gel’s network structure is obtained by aging the colloidal particles, and PLNPs are finally prepared by drying and high-temperature calcination. PLNPs synthesized by the sol-gel method are generally nanoscale and have excellent afterglow properties. In 2013, Abdukayum and his colleagues synthesized Cr^3+^, Pr^3+^ co-doped Zn_2.94_Ga_1.96_Ge_2_O_10_ nanoparticles by the citrate sol-gel method [87]. It was found that the PLNPs exhibited bright near-infrared (NIR) emission in a bio-transparent window, featuring an extraordinarily long afterglow duration exceeding 15 days. Moreover, they can be conjugated into peptide biobricks, which was expected to achieve long-term low-toxic targeted tumor imaging in vivo. In addition, by adjusting the reactant ratio, calcination temperature, pH of the sol, and raw material composition, PLNPs can be optimized and modified to achieve the regulation of PLNP particle size, morphology distribution, and optical properties [88,89]. In 2021, Zhu et al. synthesized Zn_2+*x*_Ga_4-2*x*_Sn*_x_*O_8_:0.5%Cr^3+^ (*x* = 0–0.4) NIR-PLNPs via the sol-gel method. The luminescence intensity of PLNPs was effectively regulated by changing the doping amount of Sn^4+^ [90]. It is found that the higher the x value is, the stronger the afterglow signal is. When *x* = 0.5, the afterglow intensity is the best. Although the sol-gel method has certain advantages, the obtained PLNPs still have high agglomeration and irregular morphology. The main reason is that the growth of nanoparticles during high-temperature calcination is uncontrollable, which undoubtedly limits the further application of PLNPs in the biomedical field.

**Table 2:** Comparison of synthesis methods of PLNPs.

	Preparation Method	Reaction Condition	Particle Size	Afterglow Time	Advantage	Disadvantage	Ref.
Solid Phase	High-Temperature Solid-State Method	High Temperature (˃1000 °C)	Micron scale	Long	Simple operation, low cost, superior afterglow properties	High temperature, large particles, irregular shape, easily caking, lacks control	[91,92,93]
Liquid Phase	Hydrothermal Method	Aqueous solution, high temperature and high pressure	Nano to micron scale	Short	Relatively mild, good dispersion, controllable shape and size	High facility request, low yield, long reaction time, low performance	[94,95,96]
	Sol-Gel Method	Gel, high-temperature calcination	Nano to micron scale	Mezzo	Small size, good uniformity and performance, tunable	High cost, complex craft, highly reunited irregular shape, limited application	[97,98,99]
	Template Method	Using MSNs as templates, room temperature	Nano scale	Mezzo	Mild, small size, avoids reunions, tunable	Single type of template, poor dispersion	[100,101,102]

**Figure 4:**
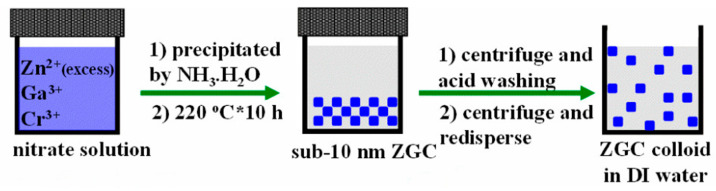
Hydrothermal synthesis of PLNPs [105]. Copyright 2015 *Journal of the American Chemical Society*.

In contrast to alternative synthesis approaches, hydrothermal synthesis typically yields PLNPs through chemical reactions conducted in a specialized high-pressure reactor. Throughout the reaction process, we need to provide a high-pressure and hermetically sealed environment, which promotes decomposition between solid precursors [103]. Moreover, the hydrothermal method can effectively control the reaction parameters, including temperature, concentration, reaction time, and PH, which makes it possible to synthesize highly crystalline PLNPs under relatively mild conditions. Importantly, PLNPs synthesized by hydrothermal synthesis have ultra-small particle sizes and are easy to surface modify [104]. At present, researchers have found that surfactants with specific functional groups can promote the success of hydrothermal synthesis and contribute to biological applications, including oleic acid, ethylenediaminetetraacetate, or polyethylenimine. In 2015, Li et al. reported ZnGa_2_O_4_Cr_0.004_ PLNPs with sub-10 nm dimensions, which can be directly synthesized in aqueous solution by the hydrothermal method (Figure 4). These nanocrystals not only have an ultra-small size but also have intense PL characteristics under red light excitation and are easily dispersed [105]. Although hydrothermal methods have great advantages in terms of particle size, dispersion, and functionalization, they still suffer from the drawbacks of weak afterglow intensity and short afterglow duration, which need to be improved upon by further research. Furthermore, they also have the problems of high equipment requirements, low yield, and difficulty in large-scale production, which hinders the development of PLNPs.

**Figure 5:**
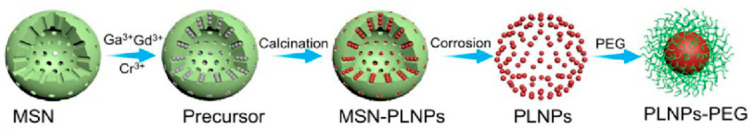
The schematic diagram of PLNP synthesis by the template method [107]. Copyright 2021 *ACS Nano*.

The template method is used to deposit the precursor into the pore or surface of the template by physical and chemical means to achieve precise control of the morphology, size, and structure of PLNPs. Mesoporous silica nanoparticles (MSNs) are commonly used as templates for PLNP synthesis. Due to their stable mesoporous structure, ultra-high specific surface area, and good biocompatibility, PLNPs synthesized by MSN templates are widely used in drug delivery and medical treatment. In addition, loading PLNPs into the pores of mesoporous silica can effectively avoid particle agglomeration and excessive growth, increase the specific surface area, and lay the foundation for further surface modification and drug loading [106]. In 2022, Li et al. [107] used MSN as a template to prepare PLNPs with ultra-small size, ultra-long persistent luminescence, and excellent dispersion properties and used them for afterglow/magnetic resonance dual-mode imaging and in vivo kidney clearance, effectively solving the problem of PLNP size and body gap regulation in biomedicine. Figure 5 is the synthesis diagram of the process. However, up to now, there has only been one kind of template that can be used to synthesize PLNPs, which greatly limits the development of materials. Moreover, the high-calcination-temperature stage of the template method may destroy the surface functional groups, resulting in poor accumulation and poor dispersion of PLNPs. Therefore, the synthesis of long afterglow materials still needs to be continuously explored to develop an ideal technology to achieve the perfect size, shape, and afterglow performance and achieve industrial production.

### 2.3. Luminescence Mechanism of PLNPs

Due to the differences in composition and structure, different types of PLNPs show their own unique afterglow mechanism. In-depth analysis of its luminescence mechanism is helpful to the real theoretical basis of rammed materials research and provides key theoretical support for the design and construction of new PLNP systems. In the early days, the understanding of the mechanism for PLNPs was limited. Since the discovery of high-performance SrAl_2_SO_4_ co-doped with Eu and Dy, researchers have gradually begun to explore the intrinsic principles of PLNPs, while many models have been proposed [108]. However, there is still a lack of a unified theoretical model to explain all afterglow phenomena due to the limitations of analytical techniques and the complexity of energy level structures. The most common understanding of PLNPs is that their afterglow phenomenon is completed by luminescent centers and trap centers, which can be divided into four stages: excitation, storage, release, and recombination [109,110]. The center of luminescence determines the wavelength of the afterglow emission, which is typically lanthanide rare earth ions or transition metal ions. The center of trap determines the duration of the afterglow emission and the initial brightness, which is generally an intrinsic defect within the material or a point defect formed during the substitution process. Based on the consideration of luminescence centers, energy band theory, electron migration, carrier types, and other knowledge, in the following, we review several prevailing theoretical models to explain the afterglow phenomenon of PLNPs.

**Figure 6:**
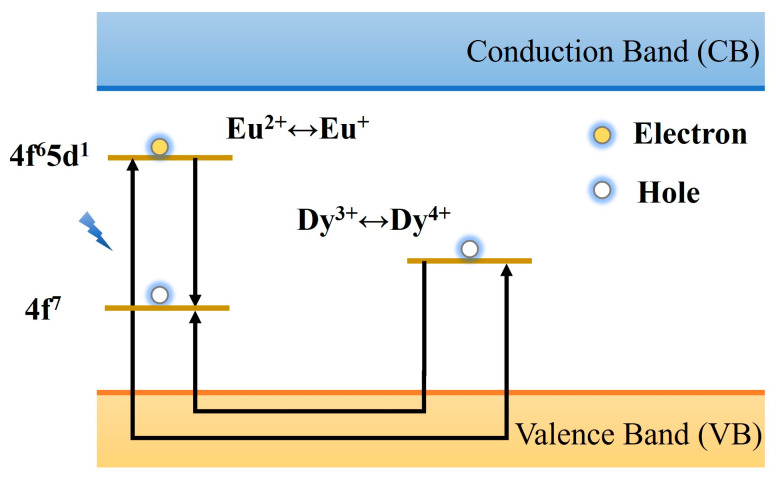
SrAl_2_O_4_: Eu^2+^, Dy^3+^ hole transfer model.

The hole transfer model is one of the classical theoretical frameworks used to explain the afterglow luminescence mechanism of materials. It was proposed by Matsuzawa et al. [32] in 1996 when studying the Eu^2+^, Dy^3+^ co-doped SrAl_2_O_4_ system. The theoretical foundation of this model originates from Abbruscato et al. [111], who utilized the Hall effect to confirm the carrier nature in SrAl_2_O_4_: Eu^2+^ PLNPs, demonstrating that the dominant carriers are hole carriers. Figure 6 shows the schematic diagram of the model. When the material is excited by ultraviolet light or visible light, the 4f^7^ ground state electron of the luminescent center Eu^2+^ transitions to the 4f^6^5d^1^ excited state, and at the same time, holes are generated in the 4f orbital, resulting in the conversion of Eu^2+^ to Eu^+^. The generated holes migrate to the valence band (VB) and are captured by the adjacent Dy^3+^ ions, which are oxidized to Dy^4+^, forming a hole trap with an energy level depth of about 0.65 eV. Owing to the shallow trap depth, thermal perturbation at room temperature can induce the escape of trapped holes. These escaped holes then migrate through the valence band to combine with electrons in Eu^+^, facilitating their transition from the excited state (Eu^+^) to the ground state (Eu^2+^) while releasing energy to generate characteristic afterglow luminescence. The role of Dy^3+^ is considered to be to prolong the carrier lifetime by regulating the capture and release kinetics of holes, thereby significantly improving the brightness and duration of afterglow. Although the early model verified the hole migration process through electron spin resonance and photoconductivity tests and reasonably explained the optimization effect of Dy^3+^ co-doping on the afterglow performance, its core hypothesis is significantly controversial. One controversy is that Qi et al. [112] characterized the material system by X-ray absorption near edge structure (XANES) and failed to detect the characteristic signals of Eu^+^ and Dy^4+^. The second is that Clabau and his colleagues calculated the energy level difference between the ground state and the valence state by X-ray photoelectron spectroscopy to be about 3 eV. This value conflicts with the shallow trap depth postulated by the model, making it difficult to support the effective release of holes at room temperature [113]. In this case, the hole transfer model is being gradually replaced by other more perfect models, but its pioneering role in the study of long afterglow mechanisms is still widely recognized.

**Figure 7:**
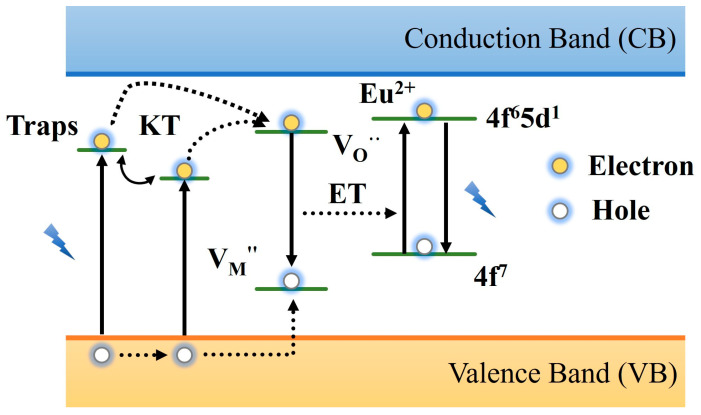
Aitasalo two-photon model.

The two-photon mechanism represents a new theory that emerged subsequent to the hole transfer model. In 2006, it was proposed when Aitasalo studied the MAl_2_O_4_: Eu^2+^, RE^3+^ (M = Ca, Sr, Ba; RE = Dy) system, which provides important support for explaining the high-energy emission phenomenon under low-energy excitation [114]. The model believes that the rare earth ion RE^3+^ does not replace M^2+^ in the matrix and instead introduces a negatively charged cation vacancy as a hole trap, while the oxygen vacancy is used as an electron trap. When the material absorbs two photons at the same time, the electrons in the VB directly transition to the trap energy level, forming an electron–hole pair. Subsequently, the electrons are trapped by oxygen vacancies, and the holes are trapped by metal cation vacancies under thermal perturbation. After stopping excitation, electrons and holes recombine and transfer energy to the luminescent center Eu^2+^, resulting in the afterglow phenomenon, as shown in Figure 7. The innovation of the model is that it breaks through the limitations of the traditional Matsuzawa model on excitation light energy. By introducing the synergistic effect of cation vacancy and oxygen vacancy, the regulation mechanism of RE^3+^ co-doping on the charge balance and trap density is clarified. However, this model still faces some controversies. One is that two-photon absorption often requires high-density light excitation, and the energy density of ordinary light sources has difficulty meeting the absorption threshold, resulting in doubts about its feasibility in practical applications. The second is that the model involves the separation, recombination, and energy transfer of various carriers. It requires a high degree of matching of each energy level, and the carriers move as expected, which is difficult to meet at the same time. Although the two-photon model has some shortcomings, the model provides a theoretical basis for the defect engineering design of PLNPs, deepens the understanding of its mechanisms, and also provides a diversified strategy for the design of high-performance materials. Future research needs to be continuously optimized in trap engineering, wavelength regulation, and cross-scale characterization techniques to promote the development of PLNPs in biomedical applications.

**Figure 8:**
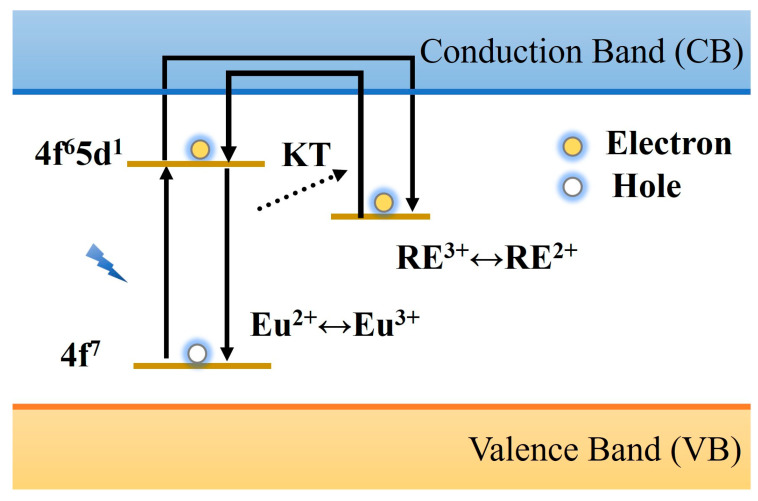
Lanthanide element trap model.

The lanthanide trap model was proposed by Dorenbos et al. [115,116] when they studied the afterglow properties of Sr_2_MgSi_2_O_7_ materials containing Eu^2+^ and Dy^3+^. They suggested that the source of electrons was excited from the doped lanthanide rare-earth ions rather than being generated from the valence band. When an electron is excited, it enters the conduction band and is trapped by the RE^3+^ ion, hence RE^3+^ is known as an electron trap, and this theory is also called the Dorenbos electron model. Thus, taking Sr_2_MgSi_2_O_7_: Eu, Dy as an example, the electrons on 4f^7^ will transition to the excited state 4f^6^5d^1^ when irradiated by the excitation light source, which makes Eu^2+^ change into Eu^3+^. After thermal perturbation, the electrons in the excited state level enter the conduction band and become free electrons, which are captured by Dy^3+^ and transformed into Dy^2+^. After the excitation stops, the electrons are released and re-enter the conduction band under the action of thermal disturbance, where they recombine with the holes of Eu^3+^ to produce the characteristic afterglow luminescence of Eu^2+^, as shown in Figure 8. The advantage of this model is that all the cation valence states involved in the process may be realized in the natural environment, but the afterglow phenomenon caused by single rare earth ion doping cannot be explained [117]. Therefore, the afterglow mechanism of PLNPs still needs further study.

**Figure 9:**
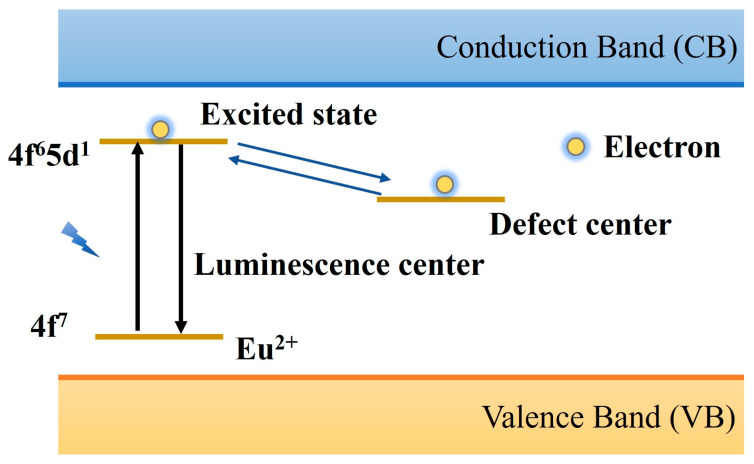
Tunneling effect model.

When the Dorenbos electron model was proposed, Clabau et al. [118] summarized and reviewed previous studies and proposed a tunneling effect model, which provided a new perspective for understanding the luminescence mechanism of PLNPs. Different from the existing models, they believe that the electron transfer does not necessarily occur through the conduction band but may move directly between the luminescence center and the trap center, as shown in Figure 9. At high temperatures, SrAl_2_O_4_: Eu^2+^, Dy^3+^ produced luminescence, but the photoconductivity test did not show the release of free carriers. There is no obvious change in the spectral shape of the material before and after doping, indicating that Dy^3+^ doping does not change the characteristics of the original trap center but plays a stabilizing role in the oxygen vacancies of the intrinsic defects in the material matrix. The results of this study imply the birth of a new electron transfer mechanism, which is realized by moving between the luminescence center and the trap center. This view provides new insight into the luminescence mechanism of PLNPs.

In addition to the above theoretical models, in recent years, researchers have proposed other models, such as the site coordinate model, the oxygen vacancy model, the electron transfer-tunneling model, the hole transfer-tunneling model [25,119], etc. These mechanisms can only explain the afterglow luminescence phenomenon of specific PLNPs and cannot be applied to all PLNP systems. Therefore, researchers need to continue in-depth research to grasp the two key points of the type of traps, the specific transmission path of trap capture and release carriers, clarify the principle, and provide clear theoretical guidance for the design and development of new PLNP systems.

## 3. Application of PLNPs in Biological Detection

Fluorescence detection technology is one of the most prevalent analytical methods in modern scientific research. It has the characteristics of high sensitivity, rapid response, simple operation, and low cost, and thus it plays a significant role in the public health and biomedical fields [120]. Over the years, a variety of optical materials have been devised and synthesized, including fluorescent dyes, upconversion nanoparticles, and quantum dots, which aims to cater to the demands of fast, instantaneous, and in situ assays. Table 3 compares the main characteristics between them. However, these traditional optical materials are often interfered with by autofluorescence, scattered light, and photobleaching of biological matrices, resulting in the weakening or even disappearance of fluorescence signals, which affects the accuracy and reliability of detection [121]. Therefore, the exploration and development of more efficient and more sensitive optical materials for fluorescence detection technology has received extensive attention from researchers.

As a distinctive material that can be capable of sustained luminescence even after excitation ceases, PLNPs not only effectively solve the problem of background signals and suppress interference from autofluorescence and scattered light, but they also have a long afterglow life and high signal-to-noise level. They are the main optical materials for a new generation of non-autofluorescence detection. At the same time, PLNP-based fluorescent probes and sensors not only have good biocompatibility and stability but also can specifically interact with biomarkers in the face of complex biological environments, with active and passive targeting capabilities, showing high selectivity and significant sensitivity, promoting the development of PLNPs in biological detection and monitoring [25,134]. In the following, we will summarize some application progress of PLNPs in biomedical detection according to the different types of biological detection materials.

### 3.1. Tumor Marker Detection

Cancer poses a severe threat to human health. It not only brings great physical and mental pain to patients but also significantly reduces the quality of life of patients. The development of cancer is occult, and its pathological changes often begin at the molecular level, which occurs quietly before the clinical symptoms are fully revealed. According to statistics, the incidence and mortality of cancer patients are high every year. Most cancer patients are diagnosed in the middle or late stage and miss the best time to treat [135]. Therefore, early screening and accurate identification are particularly important in cancer prevention and control strategies, which constitute the key links of the three-level cancer prevention system. Using advanced diagnostic techniques to accurately detect tumor markers (proteins, enzymes, nucleic acids) can detect abnormalities in the initial stage of disease development in time, thereby significantly improving the treatment effect, improving the prognosis of patients, and reducing medical costs. This has important practical significance for improving the overall level of cancer prevention and treatment.

Researchers have developed various analytical techniques to detect tumor markers, such as fluorescence immunoassay, Raman spectroscopy, optical biosensor probes, etc. [136,137]. Some of these techniques use fluorescent materials to label antigens or antibodies and then specifically bind to tumor cell markers to achieve the purpose of detection by analyzing fluorescent signals. However, the fluorescent materials used in these techniques are highly susceptible to the autofluorescence of the organism and exhibit sub-optimal sensitivity. Based on this, the fluorescence resonance energy transfer (FRET) process with PLNPs as energy donors can not only effectively solve the shortcomings of traditional technologies and skillfully overcome the interference of the fluorescence of the biological matrix caused by in situ excitation but also obtain a high signal-to-noise ratio and selectivity [138,139]. Therefore, PLNPs are expected to become potential alternative materials to make up for the shortcomings of traditional technologies.

**Figure 10:**
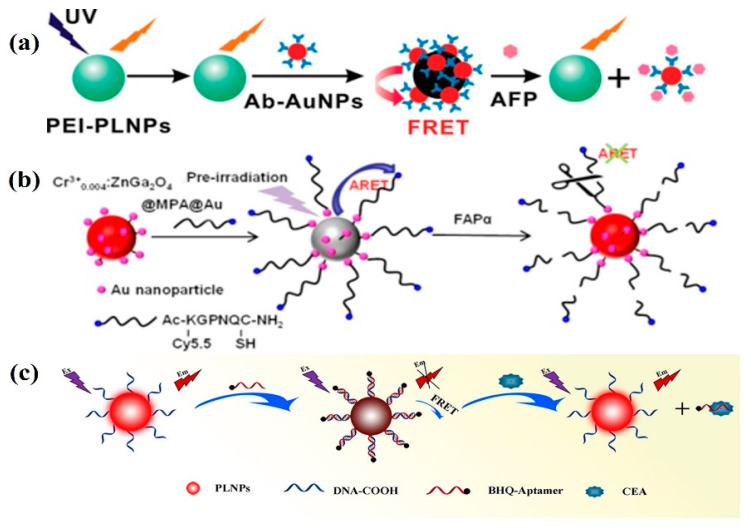
(**a**) FRET system construction and AFP detection diagram [141]. Copyright 2010 *Journal of the American Chemical Society*. (**b**) Cr^3+^ _0.004_: ZnGa_2_O_4_ @ MPA @ Au as an ARET sensor for FAP-α [142]. Copyright 2018 *ACS Sensors*. (**c**) Schematic diagram of CEA detection by FRET method based on PLNPs [143]. Copyright 2022 *Analytica Chimica Acta*.

Alpha-fetoprotein (AFP) is a key serum marker for hepatocellular carcinoma (HCC). The increase in AFP level in serum is closely related to the rapid growth of HCC cells, cirrhosis, chronic active hepatitis, and carbon tetrachloride poisoning [140]. Detection of serum AFP levels in cancer cells can be monitored in real time for the early diagnosis and treatment of liver cancer to create better conditions. In 2011, Yan et al. designed a highly sensitive and specific sustained luminescence nanoprobe. The nanoprobe is composed of polyethyleneimine-coated PLNP (PEI-PLNP) as an energy donor and AFP antibody-modified gold nanoparticles (Ab-AuNPs) as an acceptor coupled to each other for the detection of AFP in serum samples and for the detection of AFP excreted during the growth of cancer cells [141]. In this study, Yan used a fluorescence resonance energy transfer (FRET) inhibition method to induce a disruption of the FRET system in the absence of in situ excitation, which ultimately leads to the appearance of PL due to competition between AFP and PLNPs for Ab-AuNPs (Figure 10a). In addition, the PLNPs were composed of Eu^2+^ and Dy^3+^ doped Ca_1.86_Mg_0.14_ZnSi_2_O_7_, whose PL emission spectra had the greatest overlap with the absorption spectra of the Ab-AuNP couplings, and thus the greatest FRET efficiency was obtained. This study not only provides a reliable time window for AFP background-free detection but also opens up a new design for high-sensitivity and background-free biosensor nanoprobes based on PLNPs.

In addition to AFP detection, fibroblast activation protein alpha (FAP-α) and carcinoembryonic antigen (CEA) are also important biomarkers in cancer diagnosis. FAP-α is a cell surface glycoprotein expressed in more than 90% of human epithelial tumors including breast cancer, ovarian cancer, bladder cancer, colorectal cancer, and lung cancer stromal fibroblasts but not in normal fibroblasts and other normal tissues [144]. In 2018, Wang et al. [142] used Au nanoparticle-modified Cr^3+^_0.004_: ZnGa_2_O_4_ as a donor and Cy5.5-KGPNQC-SH as an acceptor to determine the content of FAP-α in living cells by afterglow resonance energy transfer (ARET), as shown in Figure 10b. The afterglow signal of the donor is quenched by the ARET between the donor and the acceptor, but when the system encounters FAP-α, the KGPNQC polypeptide sequence is specifically cleaved, thereby blocking the ARET process, and the afterglow signal of the donor is restored. The determination of FAP-α based on ARET has high sensitivity, a low detection limit, and good anti-interference ability, which provides a new idea for the detection of tumor markers. In 2022, Pan and colleagues developed a fluorescent ligand sensor based on near-infrared PLNPs, which enables accurate detection of CEA in pleural effusion via FRET-mediated quenching and recovery mechanisms (Figure 10c). Owing to the long afterglow lifetime of PLNPs and time-resolved fluorometry, the background interference from the autofluorescence of pleural effusion samples was effectively suppressed, while extraneous increases in background measurement values across different samples were ruled out [143]. The detection results revealed that the measurement accuracy for CEA levels in the cancer group and benign group was extremely high, with a detection limit as low as 0.0851 pg mL^−1^.

In addition, using a similar principle, the FRET system combined with ratiometric fluorescence detection or time-resolved Förster resonance energy transfer (TR-LRET) can be used to detect prostate-specific antigen PAS [145], miRNA-21 [146], glutathione GSH [147], etc. These research advances can promote rapid and non-invasive screening of tumors in the context of rising cancer incidence, achieve labeling and tracing of cancer cells, and provide valuable insights for future clinical treatment.

### 3.2. Detection of Bioactive Substances

Bioactive substances are organic compounds with physiological activity. They are involved in a series of physiological processes, including cell signal transduction or nerve transmission. Thus, they can directly or indirectly regulate the physiological functions of organisms and affect life activities. In recent years, researchers have actively explored molecular biology techniques and developed various biomolecule-specific detection methods, especially optical analysis techniques based on PLNPs, which greatly broaden the application of bioactive substances in biological detection and disease prevention and treatment. In 2014, Tang et al. [148] reported a novel CoOOH-modified PLNP nanoprobe for the detection of ascorbic acid (AA) in living cells and organisms. PLNPs can be used as a photoluminescence unit, which is composed of Sr_2_MgSi_2_O_7_: Eu, Dy nanoparticles with persistent afterglow properties, and CoOOH nanosheets are used as recognition units and quenchers. Based on the specific reaction between AA and CoOOH, the absorption and emission spectra of PLNPs have a strong overlap. At the same time, CoOOH effectively quenched the luminescence of PLNPs through FRET effect, but in the presence of AA, CoOOH was reduced to Co^2+^, and the luminescence of PLNPs was restored. Finally, the nanoprobe shows a high selectivity and instantaneous response, which can effectively avoid the background noise generated by in-situ excitation and the light scattering of the biological matrix, providing an effective platform for detecting active substances in cells and organisms.

**Figure 11:**
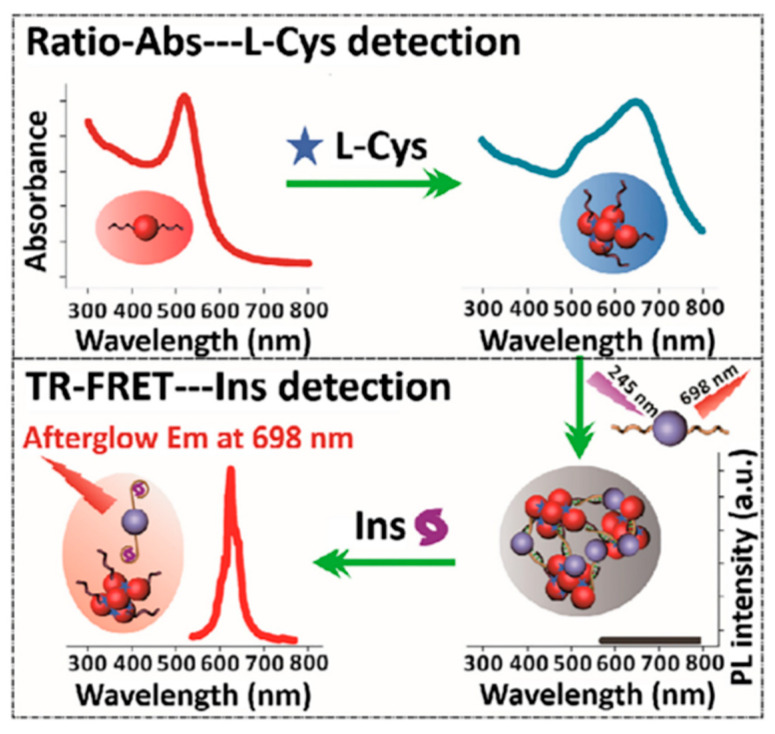
Detection based on ratiometric absorption of AuNPs and PLNPs and TR-FRET dual nanoplatform [150]. Copyright 2018 *Nanoscale*.

High levels of insulin (Ins) and L-cysteine (L-Cys) are also typical bioactive substances. They not only play a synergistic role in the life activities of organisms but also are closely related to diabetes. Ins can directly participate in blood glucose metabolism, promote glucose uptake, and inhibit liver glycogen decomposition; L-Cys can be used as a stabilizer to indirectly maintain insulin secretion and prevent protein oxidative denaturation [149]. In addition, the lack of L-Cys may also affect human growth retardation, leading to osteoporosis, Alzheimer’s disease, and other diseases and complications. Therefore, in order to effectively and continuously identify Ins and L-Cys, it is of great significance to develop specific detection of bioactive molecules. In 2018, Li et al. reported a dual-signal nanoplatform based on L-Cys-mediated ratiometric absorption and TR-FRET between gold nanoparticles and PLNPs [150]. With this platform, the researchers achieved high-throughput sequential detection of L-Cys and Ins without matrix interference (Figure 11). L-Cys can induce the aggregation of AuNPs, resulting in a significant red shift of the absorption peak from 520 nm to 660 nm, which enables sensitive detection of L-Cys through ratiometric absorption signals. The creation of the TR-FRET resulted in the spectral overlap between the emission wavelength spectrum of PLNPs and the absorption band of the assembled AuNPs, resulting in PL quenching, which was then reactivated by the strong interaction between Ins and the aptamer. The dual-signal nanoplatform has been successfully applied to the sequential determination of L-Cys and Ins in serum, which can quickly screen samples, shows excellent linearity and precision, and provides new ideas for the detection of other bioactive substances.

In addition to the bioactive substances mentioned above, hemoglobin (Hb) and the neurotransmitter dopamine (DA) also play crucial roles in physiological activities of organisms. Hb is capable of transporting molecular oxygen from the lungs to other tissues or organs, providing energy for biological metabolism. Abnormal Hb levels are closely associated with certain diseases [151]. Therefore, developing simple and accurate analytical techniques for Hb to assess and prevent certain diseases is of great necessity. In 2020, Liu et al. [152] develop ZnGa_2_O_4_: Cr^3+^ PLNPs with NIR emission properties as a label-free probe for highly sensitive and selective hemoglobin (Hb) detection. The study demonstrated that leveraging the Hb-induced dynamic quenching process, the Hb concentrations detected by this nanoprobe corresponded well with the reported levels obtained via other methods such as fluorescence spectroscopy and enzyme-linked immunosorbent assay (ELISA), confirming the probe’s applicability in detection. Moreover, the label-free probe in this work exhibited advantages of simple operation, rapid response, and ultra-sensitivity. The NIR detection window facilitates high penetration depth in complex biological samples, thereby opening new opportunities for detecting other bioactive molecules. Dopamine (DA) is an active substance essential for regulating the physiological functions of the central nervous system. Dysregulation of DA is associated with the development of Parkinson’s disease and schizophrenia [153]. The utilization of long-afterglow detection probes free from background fluorescence interference can significantly enhance detection sensitivity, thus providing support for clinical diagnosis and subsequent treatment. In 2022, Li et al. [154] combined Cr^3+^-doped ZnGa_2_O_4_ ultra-small nanoparticles with layered porous zeolite imidazole framework-8 to develop a multi-functional background-free sensing platform to detect DA by the signal quenching strategy. The proposed nanoplatform leverages superior persistent luminescence, demonstrating exceptional sensitivity and selectivity across the range of 0.0025–75 μM, with a detection limit as low as 0.0010 μM. This novel approach facilitates the development of PLNP-based biosensors and their practical implementation in clinical analysis.

**Figure 12:**
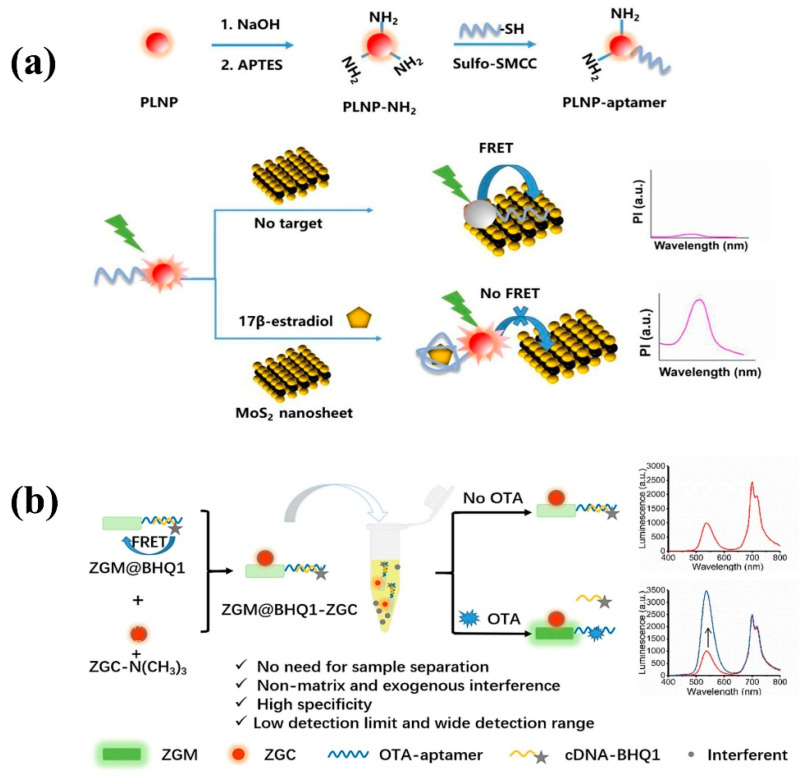
(**a**) Detection of 17β-estradiol by FRET-based aptamer-functionalized persistent luminescent nanoparticle sensor [155]. Copyright 2022 *Food Chemistry*. (**b**) Dual-color PLNPs ratiometric aptasensor for OTA detection [156]. Copyright 2023 *Food Chemistry*.

The detection of bioactive substances is also the core link of food supervision. Combined with rapid screening and detection technology, food safety capabilities can be further improved to ensure the health of consumers. Take the estrogen 17β-estradiol as an example. The illegal injection of 17β-estradiol promotes the growth of aquaculture, poultry, and livestock, which will not only cause the hormone content in food to exceed the standard and cause serious disorders of the human endocrine system but also lead to environmental pollution. Therefore, the detection of 17β-estradiol is particularly important. In 2022, Zhang et al. designed a near-infrared PLNP aptamer sensor for autofluorescence-free detection of 17β-estradiol [155]. The sensor uses 17β-estradiol aptamer-coupled NIR PLNPs as an energy donor and MoS_2_ nanosheets as a quencher to jointly construct a FRET pair (Figure 12a). When 17β-estradiol is present as a target molecule, the FRET effect can be effectively inhibited by competing with MoS_2_ to bind aptamers, thereby activating the emission of PLNPs. The recovered PLNP phosphorescence signal avoids the interference of the matrix’s own fluorescence and exhibits high selectivity and sensitivity to 17β-estradiol, which is suitable for complex sample systems. This method also provides a general strategy for rapid and specific autofluorescence-free detection. Furthermore, certain toxic active substances may also be present in food. Early detection of potential toxic contamination in food can prevent acute poisoning or chronic hazards caused by toxins, thereby ensuring food safety. In 2023, Zhao et al. [156] reported an exogenous interference and autofluorescence-free ratiometric aptamer sensor based on two-color PLNPs for the accurate detection of ochratoxin A (OTA). OTA is a bioactive substance mainly produced by Penicillium and Aspergillus. It has strong carcinogenicity, teratogenicity, and immunotoxicity. It is widely found in coffee, barley, wheat, fruits, vegetables, and other agricultural products and their derivatives. If the content of OTA exceeds the standard, it will cause serious harm to the human body. Therefore, it is of great significance to construct a rapid, accurate, and sensitive OTA detection technology and strict restriction standards in food [157]. In this work, the experimental team combined green-wavelength-emitting ZnGeO: Mn nanoscale long-afterglow materials with OTA aptamers and BHQ1-modified complementary bases as detection and specific recognition probes. Red-wavelength-emitting functionalized ZnGaGeO: Cr PLNPs were employed as reference probes. A ratiometric aptasensor was constructed via electrostatic interactions, as illustrated in Figure 12b. The developed sensor is free from interference by real-time excitation, external environments, and autofluorescence, with a detection limit as low as 3.4 pg·mL^−1^, a wide linear range of 0.01–50 ng·mL^−1^, and a precision of up to 3.1%. This study also demonstrates good versatility, holding promise for extension to the detection and analysis of other bioactive substances through replacement of aptamers and corresponding complementary strands.

Overall, bioactive substances play an irreplaceable role in maintaining human health, regulating physiological functions, and preventing chronic metabolic diseases. Traditional detection techniques such as high-performance liquid chromatography (HPLC) and mass spectrometry (MS) face bottlenecks including operational complexity, limited sensitivity, and difficulty in real-time monitoring, making them inadequate for precise detection of active components in complex matrices. The novel persistent luminescent nanoparticle PLNP detection system constructs a self-powered detection platform without a real-time excitation light source, which successfully makes up for the shortcomings of traditional technology. It not only breaks through the limitations of photobleaching and autofluorescence in traditional optical detection but also realizes the high selective capture of specific bioactive substances by surface functionalization, and the detection sensitivity can reach picomolar level. This new technological system exhibits broad application prospects in various fields, providing strong technological support for personalized nutritional intervention and precision medicine. It marks a significant leap in the research of bioactive substances from qualitative analysis to intelligent quantitative detection.

### 3.3. Pathogenic Microorganism Detection

With the acceleration of global urbanization, ecological environmental disturbances and population susceptibility have gradually increased. Some pathogenic microorganisms, relying on high-density population aggregation and frequent cross-regional mobility, can fully spread within just a few days. This not only exacerbates the risk of disease transmission but also poses a significant threat to human health security. Therefore, developing rapid, precise, and complex-sample-compatible pathogenic microorganism detection technologies has become a core demand in public health emergency management. Traditional viral detection relies on virus isolation and culture, enzyme-linked immunosorbent assay (ELISA), and polymerase chain reaction (PCR) [158,159]. However, in complex biological samples (such as serum and biofilm matrices), detection sensitivity, specificity, and high-throughput screening capabilities are still challenged by inherent limitations. In recent years, the development of new detection platforms based on PLNPs that simultaneously incorporate molecularly imprinted polymer technology (MIP), time-resolved fluorescence (TRP), image algorithm coding, and other technologies has provided an innovative path for solving traditional technical problems and has also led to an intelligent detection of pathogenic microorganisms [160,161,162].

**Figure 13:**
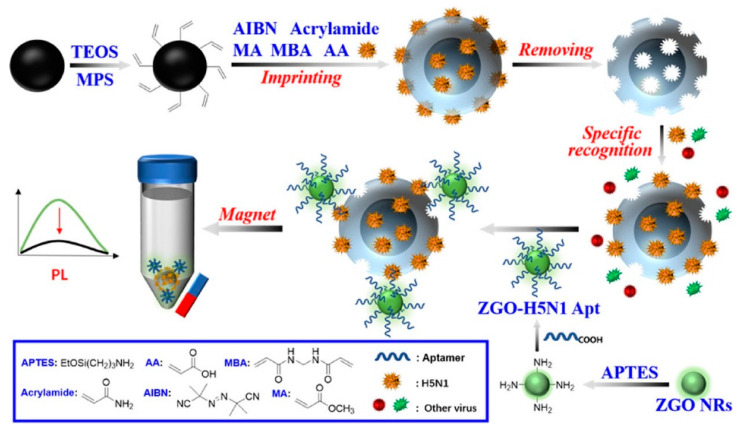
MIP-aptamer sensing for H5N1 virus detection [163]. Copyright 2022 *ACS Applied Materials & Interfaces*.

In 2022, Cai et al. [163] exploited the luminescence attenuation effect of PLNPs to circumvent autofluorescence interference in complex biological samples while developing a non-autofluorescent MIP-aptamer sensor for H5N1 virus detection (Figure 13). In this work, the researchers used magnetic Fe_3_O_4_ as the imprinting carrier to construct the MIP network of the H5N1 virus template and combined it with aptamer-functionalized Zn_2_GeO_4_: Mn^2+^ PLNPs to form a magnetic carrier-virus-aptamer probe sandwich structure. Upon recognizing the H5N1 virus, it produces strong persistent luminescence signal changes and finally attains an extensive detection range and desirable detection limit (0.0128 HAU mL^−1^). The MIP-aptamer sensing integrates the advantages of ZGO PLNPs, MIP, and an aptamer, serving as a universal platform for highly selective and sensitive detection. Furthermore, by changing the imprinted template molecule and aptamer, it can be easily extended to detect other analytes, providing a new strategy for the non-autofluorescence determination of targets in complex biological samples.

**Figure 14:**
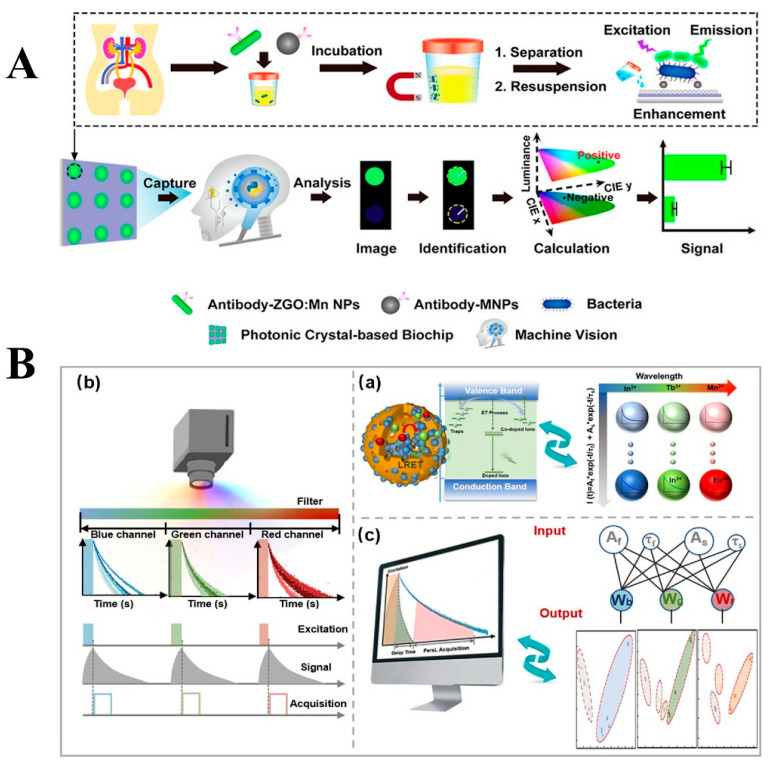
(**A**) is based on the machine vision diagnostic system for detecting bacteria in urine [164]. Copyright 2021 *Nano Letters*. (**B**) A specially constructed time-resolved fluorescence encoding technique for coronavirus detection. (**a**) Strategy for PL wavelength encoding based on energy transfer mechanisms. (**b**) Schematic illustration of the time-resolved PL method. (**c**) Decoding of PL barcodes using fitting parameters [165]. Copyright 2022 *Analytical Chemistry*.

In addition to achieving specific detection of multiple pathogenic microorganisms by replacing molecular imprinting templates and aptamers, the introduction of coding techniques or intelligent algorithms based on persistent luminescent nanomaterials has also demonstrated significant innovative value in highly sensitive detection of pathogenic microorganisms in complex samples. Not only does this detection approach effectively eliminate interference from biological autofluorescence by leveraging the luminescent properties of PLNPs, but it also enables multidimensional signal acquisition through coding strategies. By applying algorithms for amplification, noise reduction, and feature extraction of weak signals, this method overcomes the sensitivity bottleneck of traditional detection techniques in complex matrices, providing a novel solution that integrates anti-interference capability and data analysis capability for pathogenic microorganism detection. In 2021, Yuan et al. constructed a machine vision diagnostic system for detecting the presence of bacteria in urine samples by integrating Python image algorithms into a photonic crystal biochip based on PLNPs (Figure 14A). In this study, antibody-modified Zn_2_GeO_4_: Mn (ZGO: Mn) PLNPs could capture target bacteria by antigen-antibody specific recognition [164]. After magnetic separation and resuspension, the ZGO: Mn PLNPs showed significantly enhanced afterglow signals in photonic crystals on the biochip and were able to convert the optical signals into digital signals by a machine vision algorithm. The algorithm–biosensor combination rapidly detected bacteria presence in urine with a low detection limit and wide dynamic range, providing an economical and friendly digital diagnosis scheme for modern health management and personalized medicine. In 2022, Lv et al. [165] proposed a persistent luminescence (PersL) lifetime/color binary coding strategy for the five-fold detection of SARS-CoV-2 coronavirus, as shown in Figure 14B. Based on the FRET process, researchers have developed PLNPs with tunable PersL lifetime and emission spectrum and realized the creation of new time-encoding dimensions from seconds to minutes in three separate channels. Combined with TRP technology, the emission spectrum is captured with an appropriate time delay after ultraviolet excitation, which effectively eliminates the interference of autofluorescence or scattered light and improves the detection sensitivity. This specially constructed TRP technology integrates a time-correlated spectrometer and a custom fitting algorithm. Using the iterative least squares method, it can be decoded in PersL emission and lifetime modes and shows excellent linearity and low-level detection limits for coronavirus detection. More importantly, with a background without autofluorescence and high-fidelity resolution, PersL lifetime/color binary coding can distinguish mixtures with high specificity in practical applications, paving a new way for multiple biological applications.

Despite the remarkable advantages of PLNPs in biological detection owing to their unique optical properties (such as ultra-long luminescence lifetime, background-free interference, and good biocompatibility), the current applications of such materials still face critical technical bottlenecks. Most of the existing systems are limited to the detection of a single biomarker, which is difficult to meet the needs of simultaneous analysis of multiple indicators in complex biological samples. This single detection mode not only leads to low detection efficiency and a cumbersome operation process, but also may affect the correlation of results due to the time difference introduced by fractional detection, which cannot fully reflect the dynamic changes in biological systems. To address this challenge, the development of PLNPs with multi-wavelength emission characteristics has become an important direction for future research. Various means such as doping different rare earth ions and designing different morphologies, heterostructures, or multi-component composite systems are used to optimize and improve the materials, accurately regulate their energy level structures, and achieve synergistic emission of multiple characteristic luminescent bands, so as to achieve differential identification of multiple biomarkers in a single detection system. At the same time, by constructing a multi-dimensional data model and combining intelligent algorithms, it is expected to further break through the sensitivity limit of traditional detection technologies and show a broad application prospect for real-time dynamic monitoring of multiple targets.

## 4. Application of PLNPs in Biological Imaging

Bioimaging techniques represent one of the most commonly used methods for monitoring the structures, cellular distribution, and physiological activities within living organisms, playing an irreplaceable role in disease diagnosis, drug target therapy, and basic biological research. To obtain clear, accurate, and interference-free image information, imaging materials are of utmost importance. Optical materials used in traditional fluorescence imaging techniques mostly require continuous excitation light sources, which not only suffer from interference by background fluorescence but also pose phototoxicity issues, severely affecting the sensitivity and accuracy of imaging. In recent years, the development of PLNPs has brought about new breakthroughs in bioimaging. Following the termination of excitation, these materials can still achieve ultra-long afterglow emission through their unique luminescent mechanism. This not only effectively eliminates background signal interference but also significantly reduces photodamage, providing an innovative solution for obtaining bioimages with a high SNR. Meanwhile, precise regulation of the size, morphology, and luminescent properties of PLNPs can greatly enhance their optical stability and biocompatibility, thereby promoting their in-depth applications in bioimaging.

At present, PLNPs have been widely used in fingerprint imaging, cell imaging, and in vivo imaging to assist criminal investigation and dynamic monitoring of physiological or pathological processes in organisms. In addition, combining multimodal imaging technology and integrating the advantages of different imaging modes, it can provide multi-dimensional information support for the accurate diagnosis and treatment of complex diseases, fully demonstrate the broad prospects of PLNPs in the field of bioimaging, and is expected to promote key breakthroughs in related technologies.

### 4.1. Fingerprint Imaging

A fingerprint, as a unique biomarker system of human skin, is regulated by genetic genes to form unique morphological differences. It not only contains macroscopic phenotypic characteristics such as epidermal texture and sweat gland distribution but also carries multi-dimensional biomolecular information such as exogenous residues, epidermal metabolites, and sweat gland secretions. This biological attribute from microscopic molecular mechanism to macroscopic phenotypic expression makes it a natural information base for analyzing individual biological characteristics. Traditional fingerprint imaging technology mainly relies on physical morphological characteristics. However, in the face of invisible fingerprints such as latent fingerprints (LFPs), it is often difficult to meet the needs of high-precision biological information acquisition due to the problems of complex actual samples and background signal interference, resulting in blurred imaging, low SNR, and sensitivity. The introduction of long afterglow materials provides a new way to break through this technical bottleneck. Based on the advanced optical probe of nano-persistent PLNPs, it can specifically bind to biomolecules in fingerprints (such as amino acids, electrolytes, proteins, DNA, and drug residues in sweat) and enhance the visibility of latent fingerprints by fluorescence labeling. Its unique excitation/emission spectrum can effectively eliminate complex background interference, collect afterglow signals after the background signal disappears, significantly improve the sensitivity and resolution of bioinformation acquisition, and realize the leap from traditional body surface texture imaging to biomolecular functional imaging. It provides theoretical and technical support for the construction of a high-precision fingerprint bioinformation decoding system and highlights the frontier value and application potential of PLNPs in the field of bioimaging.

**Figure 15:**
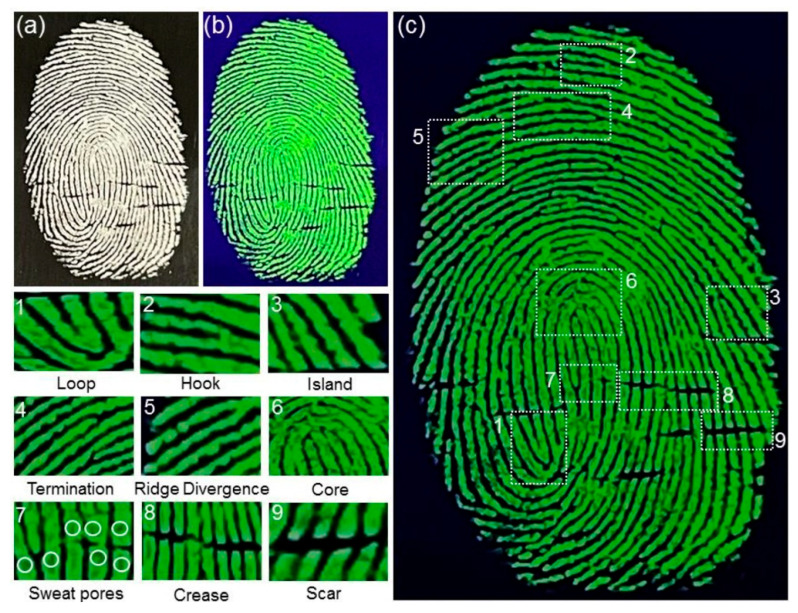
Zn_2_GeO_4_: Mn^2+^ nanoparticles for potential human fingerprint imaging on aluminum foil substrate surface. (**a**) daylight and (**b**) 254 nm UV lamp irradiation. (**c**) The sharpened and magnified images show nine kinds of details, specifically: (1) loop, (2) hook, (3) island, (4) termination, (5) ridge divergence, (6) core, (7) sweat pores, (8) crease, and (9) scar [166]. Copyright 2022 *Materials Today Chemistry*.

In 2022, Xue et al. [166] successfully prepared Mn^2+^ doped Zn_2_GeO_4_ nanoparticles (ZGO PLNPs) by solid phase reaction and applied it to human potential fingerprint imaging on the surface of an aluminum foil substrate, as shown in Figure 15. The breakthrough of this study is that the synthesized ZGO PLNPs exhibit excellent green afterglow characteristics, and their internal quantum efficiency is as high as 98.5%, which is significantly better than traditional fluorescent probes and most reported phosphorescent nanomaterials. Under the irradiation of sunlight and a 254 nm ultraviolet lamp, ZGO PLNPs can clearly reveal the multi-level detailed features of fingerprints, including macro-structures such as rings, hooks, and islands in fingerprint patterns; detailed features such as the end, bifurcation, and ridge divergence of mastoid ridge lines; and even microscopic features such as sweat pores, creases, and scars. This high-resolution imaging ability is due to the specific adsorption of fingerprint substances by nanoparticles and an efficient energy conversion mechanism. The doping of Mn^2+^ ions not only broadens the excitation spectrum range of the material but also optimizes the electron transition path through lattice distortion so that the material can produce a long-lived afterglow emission after ultraviolet excitation, effectively reducing ambient light interference. The successful application of ZGO PLNPs not only provides an efficient solution for obtaining high-quality and high-resolution fingerprint image information for impermeable substrates such as aluminum foil but also creates a new path for material evidence display based on afterglow nanomaterials.

However, the current PLNP-based fingerprint imaging technology is still in its infancy, and there are many areas that need to be optimized and improved. From the perspective of material properties, although the internal quantum efficiency of some PLNPs is extremely high, its surface defects and agglomeration may lead to rapid attenuation of phosphorescence brightness in practical applications. Especially in long-term excitation or humid environments, the afterglow intensity will decrease significantly, affecting the stability of imaging. From the analysis of the imaging mechanism, the existing methods rely on the physical adsorption of nanoparticles and fingerprint residues and lack the targeted recognition ability of specific components (such as amino acids, inorganic salts) in sweat stains, which may lead to non-specific adsorption in complex backgrounds and increase the difficulty of image post-processing. In addition, the response time of the technology is still unable to meet the needs of rapid detection in criminal investigation sites, and the portability and energy efficiency of the excitation light source also limit its application in field scenes. In view of these shortcomings, in the future, researchers can improve the dispersion and chemical stability of nanoparticles through surface modification and further improve the luminous brightness and anti-interference ability. PLNPs with multiple excitation wavelengths (such as a near-infrared excitation light source) and targeting ability to biomolecules loaded on the surface of fingerprints were developed to realize fingerprint-specific component recognition, shorten response time, reduce background noise, and improve imaging clarity and sensitivity. These improvements lay an important foundation for the realization of specific, accurate, and fast fingerprint imaging.

### 4.2. Cell Imaging

Cells are the basic unit of life activities. Labeling cells by means of imaging and accurately analyzing their structure, function, and metabolic activities constitute the cornerstone of modern life science research, which is of irreplaceable significance for revealing the mysteries of life processes, the pathogenesis of diseases, and drug development. However, the composition of the cell itself is complex, and there are a large number of organic substances inside, which can easily produce signal interference. In addition, the nanoscale and millisecond dynamic changes in the subcellular structure restrict the sensitivity and resolution of imaging. Therefore, how to break through the bottleneck of existing technology and realize multi-dimensional dynamic imaging of cells has become one of the core challenges of current research. Benefiting from the development of nano-persistent luminescent materials, the construction of PLNP-labeled probes not only effectively solves the influence of cell autofluorescence but also enhances the clarity and SNR of imaging, showing unique advantages.

**Figure 16:**
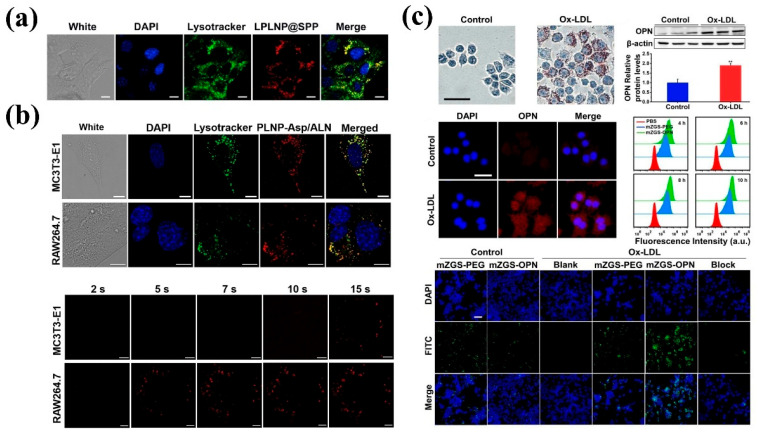
(**a**) In vitro cell imaging of LPLNP@SPP [167]. Copyright 2021 *Talanta*. (**b**) Cell imaging of PLNP-Asp/ALN [168]. Copyright 2024 *Chemical Engineering Journal*. (**c**) Construction of foam cells and in vitro fluorescence imaging [169]. Copyright 2024 *ACS Nano*.

In 2021, Zhang et al. [167] designed and synthesized a bifunctional core-shell structure of persistent luminescent polypyrrole nanocomposites (LPLNP@SPP) for cell imaging using mouse breast cancer cell 4T1 as a model. It was found that under a confocal laser scanning microscope (CLSM), 4T1 cells incubated with LPLNP@SPP showed obvious NIR persistent luminescence signals in the cytoplasm and co-localized with lysosomal markers, indicating that the composite entered the cells mainly through endocytosis and enriched on lysosomes. As shown in Figure 16a, this localization characteristic provides a basis for subsequent in vivo studies. In addition, cytotoxicity evaluation showed that the cell survival rate was more than 80% after 24 h and 48 h, which proved its good biocompatibility. In this study, the PLNPs show the characteristics of near-infrared without in situ excitation, which effectively avoids the interference of background fluorescence and improves the clarity of subcellular structure imaging. It not only clarifies the distribution of LPLNP@SPP in cells but also reveals the direct interaction between materials and cells, which provides a basis for understanding the targeting mechanism and therapeutic effect.

In addition to breast cancer cell imaging, in 2024, Wu et al. [168] reported that nanoparticles with near-infrared continuous emission and near-infrared two-photon excitation characteristics were modified by the D-Asp8 peptide and integrated with alendronate (ALN) to form a PLNP-Asp/ALN composite system for osteoclast imaging. In the experiment, the researchers used mouse embryonic osteoblast precursor cell MC3T3-E1 and osteoclast precursor cell RAW264.7 as models. Confocal microscopy showed that the target peptide D-Asp8-modified material was effectively enriched in the lysosomes of osteoclast precursor cells and produced a strong near-infrared afterglow signal within 5 s. In contrast, the signal of MC3T3-E1 cells was weak, the uptake rate was slow, and the obvious signal appeared at 15 s, as shown in Figure 16b. This work clearly reveals the selective binding ability of PLNP-Asp/ALN to osteoclast precursor cells. In particular, high-resolution imaging achieved by two-photon excitation can observe signal spots with a diameter of less than 0.74 μm, breaking through the resolution limit of traditional fluorescence imaging. In addition, the near-infrared two-photon excitation characteristics of long-persistent luminescent materials reduce light scattering and autofluorescence interference at the cellular level. Combined with targeted peptide modification, the accurate identification of low-abundance osteoclasts is realized, which provides support for the dynamic monitoring of osteoclasts and targeted drug delivery in the treatment of osteoporosis. In the same year, Shi et al. [169] focused on the early detection of atherosclerosis (AS) and developed a highly sensitive near-infrared persistent luminescence nanoprobe (mZGS-OPN) modified with OPN protein. The targeted imaging ability of the nanoprobe was verified in vitro by using a macrophage-induced foam cell model. Flow cytometry and confocal microscopy showed that compared with unmodified mZGS-PEG, the fluorescence intensity of mZGS-OPN in foam cells increased significantly with the prolongation of incubation time, while the fluorescence signal was significantly weakened after pre-incubation with the OPN antibody, confirming its ability to specifically bind to foam cells, as shown in Figure 16c. What is more, the probe distributes uniformly within cells without obvious aggregation, exhibiting extremely low toxicity, and serves as a highly specific tool for in vitro targeted imaging of foam cells during the formation of early atherosclerotic plaques.

PLNPs have shown multi-dimensional advantages in the field of cell imaging. Their ultra-long afterglow characteristics, high biocompatibility, and functional designability all provide an innovative path for traditional fluorescence imaging technology. However, current cell imaging applications still face multiple challenges. Most of the existing cell imaging is based on in vitro cultured cells or simple tissue models, which is essentially different from the complex physiological environment in real organisms. Factors such as in vivo tissue scattering, body fluid dilution, and cell metabolism can affect their luminescence intensity, resulting in significant signal attenuation during deep tissue imaging. At the same time, the targeting groups of some materials may also fail due to protein adsorption or endocytosis in long-term imaging, affecting the specific recognition ability. In addition, in vitro cell imaging achieves sub-micron resolution, but in vivo, the actual imaging accuracy may be significantly reduced due to the light scattering effect of deep tissue penetration.

Therefore, in the future, researchers need to continuously optimize and improve, develop microenvironment-responsive PLNPs, enhance the targeting and stability of materials in the complex microenvironment in vivo, and reduce non-specific adsorption and metabolic clearance; by adjusting the ion doping ratio, the design of different excitation and emission wavelengths can be realized, which can reduce the autofluorescence interference of the tissue and improve the deep penetration ability. In addition, cell imaging is a key bridge between in vitro mechanism research and in vivo precision diagnosis and treatment, and its ultimate goal is to provide reliable preliminary data support for in vivo imaging. When the targeting, stability, and biosafety of PLNPs at the cellular level are further optimized, the application value in vivo will be deeply activated. The following will focus on the application of PLNPs in living tissue imaging and explore its imaging mechanism and technological breakthroughs in the complex biological environment.

### 4.3. In Vivo Imaging

In vivo imaging, a form of biological imaging at the whole organism level, breaks through the limitations of traditional ex vivo detection. Through means such as fluorescent labeling and molecular probes, it enables real-time and dynamic visual monitoring of functional activities in living cells, tissues, organs, and even systems. This technology presents multilevel biological information ranging from microscopic molecular responses to macroscopic systemic reactions, providing an intuitive in vivo observation perspective for life science research.

**Figure 17:**
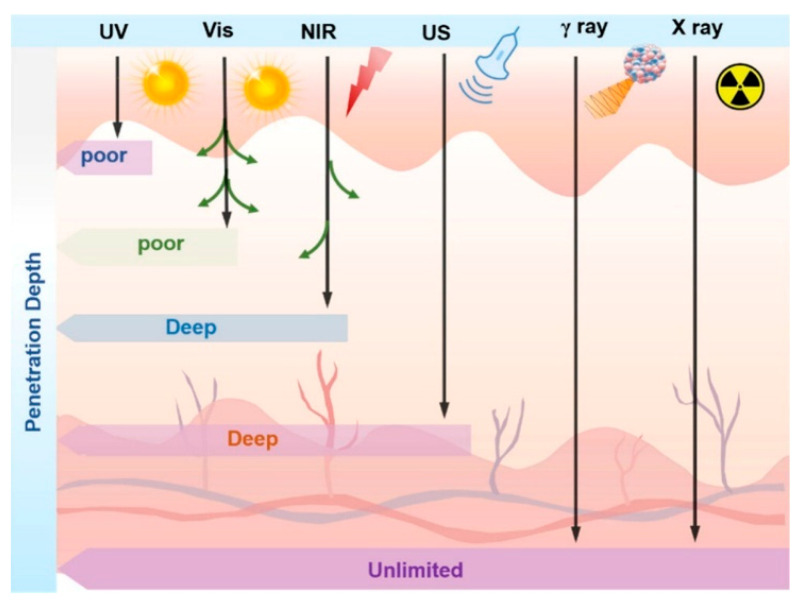
Different excitation sources have different penetration depths [172]. Copyright 2024 *ACS Applied Materials & Interfaces*.

The main characteristics of the probe material in in vivo imaging applications are as follows: (1) must be nanoparticles (size < 100 nm); (2) intense emission extending into deep red to NIR (tissue window transparency) with sustained luminescence capability; and (3) be highly functionalizable and capable of maintaining stability in biological media. In 2007, Scherman et al. published the first paper to report the use of Ca_0.2_Zn_0.9_Mg_0.9_Si_2_O_6_: Eu^2+^, Dy^3+^, and Mn^2+^ PLNPs for in vivo imaging in mice [33]. This is an example of the first generation of PLNPs used for in vivo detection without any background signal, and the afterglow can last for 1 h in vivo. Subsequently, different types of PLNPs were synthesized and applied to in vivo imaging. However, for long-term in vivo imaging, these materials have the problem of requiring ultraviolet light excitation, which affects the effect of in vivo imaging to some extent. In 2014, Richard et al. [170] prepared a new generation of a white LED repeatedly excited ZnGa_2_O_4_: Cr nano long afterglow material for in vivo imaging, which effectively solved the above bottlenecks. In 2017, Yuan et al. compared the properties of Zn_1.2_Ga_1.6_Ge_0.2_O_4_: Cr PLNPs with Ag_2_Se quantum dots and cyan-derived dyes under visible light excitation for imaging in organisms [104]. The results show that PLNPs have a higher signal-to-background ratio and sensitivity, which is attributed to the effective removal of tissue autofluorescence and light scattering interference. However, visible light excitation in in vivo imaging always shows the disadvantage of insufficient tissue penetration depth. In contrast, NIR and X-ray excitation are closer to the biological transparent window, which can obtain deeper tissue penetration (Figure 17) and broaden the application of PLNPs in the field of in vivo imaging [171,172].

**Figure 18:**
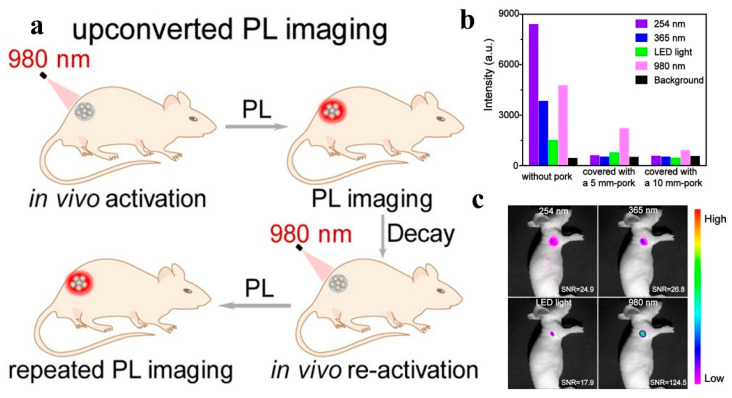
(**a**) Near-infrared light excitation bioimaging schematic diagram. (**b**) The penetration depth of different activated light sources was compared by using the meat coverage method to simulate the internal environment. (**c**) Lymphatic imaging in nude mice under 254 nm light, 365 nm light, white LED, and 980 nm light excitation [173]. Copyright 2017 *ACS Applied Materials & Interfaces*.

In 2017, Feng et al. designed a near-infrared hybrid nanocluster consisting of upconversion nanoparticles β-NaYbF_4_: Tm @ NaYF_4_ and Zn_1.1_Ga_1.8_Ge_0.1_O_4_: Cr PLNPs for biological tissue imaging [173]. In this study, Feng and his colleagues compared the imaging performance of the nanoclusters under 254 nm, 365 nm, white LED, and 980 nm light excitation (Figure 18a). When covered with a piece of pork with a thickness of 5 mm, the persistent luminescence signals of PLNPs excited by 254 nm, 365 nm, and white LED were almost undetectable. In contrast, the luminescence signal of PLNPs in 10 mm pork was still detectable under 980 nm near-infrared excitation, showing the efficient tissue penetration ability of near-infrared excitation, as shown in Figure 18b. In vivo biological tissue imaging tests further showed that the SNR of PLNPs under 980 nm excitation was as high as 124.5, which was about five times that of 254 nm excitation light, reflecting that near-infrared in vivo excitation can achieve higher imaging sensitivity, as shown in Figure 18c. In 2021, Han et al. [174] developed a CaSnO_3_: Bi nanoparticle excited by 740 nm near-infrared light. The PLNPs detected obvious afterglow signals in mice, which could maintain excellent light stability and reproducibility, showing good application potential for imaging deep tissues without background fluorescence interference.

**Figure 19:**
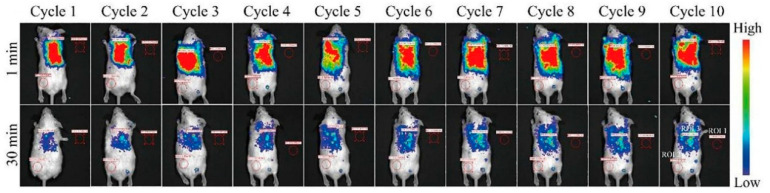
Repeated imaging of PLNPs in the liver region of mice after X-ray excitation [176]. Copyright 2021 *Chemical Engineering Journal*.

X-rays show deeper tissue penetration than near-infrared, and PLNPs excited by X-rays have broader prospects. Generally, X-ray-excited PLNPs are composed of three parts: an X-ray photon absorption center, luminescence center, and trap center. In 2020, Yang et al. [175] proposed an X-ray-excited high-gap rare earth ion Sm^3+^ doped persistent luminescent nanoparticle YPO_4_, which successfully achieved efficient deep tissue bioimaging in mice by adjusting the depth of the defect center and improving the afterglow behavior. However, the X-ray absorption efficiency often increases exponentially with the atomic number. Theoretically, a specific compound with a reasonable atomic number (such as heavy elements) as the substrate of PLNPs can improve the X-ray absorption efficiency, reduce the X-ray dose required to excite PLNPs, and avoid X-ray radiation damage to normal tissues. In 2021, Wang et al. [176] reported a low-dose X-ray-excited LaGaO_3_: Sb^3+^, Cr^3+^ long afterglow nanomaterial using La (with a higher atomic number than Zn and Li) as an X-ray absorption center. PLNPs exhibit high X-ray absorption efficiency. At the same time, co-doped Sb^3+^ ions with mismatched particle sizes were used to optimize the concentration of oxygen vacancies in the host, and the effective traps showed the best distribution and density, which improved the afterglow performance of the nanoparticles, and the afterglow life was as high as 500 h. In vivo imaging results demonstrate that the PLNPs enable repeated activation imaging in living organisms under a very low dose of 0.37 Gy X-ray irradiation, as shown in Figure 19. This strategy enriches the field of in vivo imaging research and provides useful guidance for the development of PLNPs excited by low-dose X-rays. In addition to ultraviolet, visible, NIR, and X-rays as excitation sources, in 2020, Sun et al. [177] combined the ZnGa_2_O_4_: Cr PLNPs with the tumor radiopharmaceutical ^18^F-FDG and effectively excited PLNPs through Cerenkov resonance energy transfer and ionizing radiation. Due to the high specificity of the radiopharmaceutical, nanoparticles can be selectively excited for tumor site imaging. This strategy can not only improve the sensitivity, contrast, and decay lifetime of imaging but also reduce the exposure time of patients to radionuclides.

**Figure 20:**
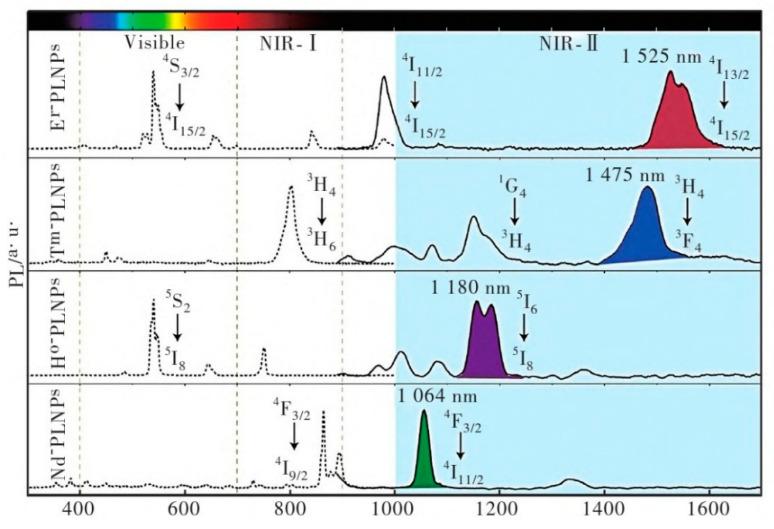
Tunable PL spectra of lanthanide-doped PLNPs after X-ray irradiation [14]. Copyright 2021 *Nature Nanotechnology*.

Although the above research effectively solves the problem of the long persistent luminescent material excitation wavelength in the application of in vivo imaging, the afterglow emission wavelength also has a profound impact on biological imaging. At present, the emission wavelengths of the reported long afterglow nanomaterials are mostly concentrated in the NIR I region (650–900 nm), and the imaging depth is limited. In contrast, PLNPs with NIR II region (1000–1400 nm) and NIR III region (1500–1800 nm) emission characteristics, as the second and third windows of organisms, can further reduce the scattering phenomenon, and the autofluorescence background noise of organisms almost disappears, which has more advantages in improving the imaging SNR, image clarity, and penetration depth. In 2021, Zhang et al. [14] reported the PLNPs emitted by the X-ray-excited NIR II region. By adding different lanthanide dopants to NaGdF_4_ nanoparticles, the emission wavelength of PLNPs has excellent tunability in the NIR II region, as shown in Figure 20. After tail vein injection into mice, it can clearly identify and image mouse blood vessels, tumors, and ureters at 2-4 mm deep tissue, showing a higher SNR and resolution. In 2023, Wang and his research team described a type of novel MgGe_0.8_Ga_0.2_O_3_: Yb^3+^ (MGGO: Yb^3+^) PLNP, which was prepared by means of the solvothermal liquid-solid solution combined with the salt microemulsion method [178]. It was discovered that substituting a portion of Ge^4+^ with Ga^3+^ ions in the MgGeO_3_ host matrix can elevate the trap density, thereby remarkably enhancing the NIR-II persistent luminescence intensity of Yb^3+^ ions. Moreover, X-rays can be used to repeatedly stimulate the PLNPs with an optical penetration length of 3.9 mm. The foregoing results demonstrate that MGGO: Yb^3+^ PLNPs hold promise as a material to be utilized within the domain of deep-tissue imaging.

In vivo imaging is not only a detection tool but also a bridge between basic research and clinical transformation. The application of PLNPs in this field is a hot topic in future research. It is necessary to explore more abundant tissue imaging objects, develop PLNPs with NIR II/III emission in various systems, and strive to improve the tissue penetration depth and sensitivity of imaging so as to make in vivo imaging move towards the direction of lower dose radiation, higher spatial and temporal resolution, and longer window monitoring.

### 4.4. Multimodal Imaging

The rapid development of precision medicine requires imaging technologies with superior sensitivity and high resolving power to achieve large-scale and cross-scale imaging so as to obtain all-round biological information. Thus far, a variety of imaging modalities have been established, including X-ray computed tomography (CT), fluorescence imaging, magnetic resonance imaging (MRI), positron emission tomography (PET), and ultrasound imaging. Each imaging mode has its own characteristics, advantages, and limitations. Multimodal imaging combines the advantages of different imaging methods; overcomes the inherent limitations of single-modal imaging technology; and provides more accurate, complete, and reliable image information for disease diagnosis and treatment.

**Figure 21:**
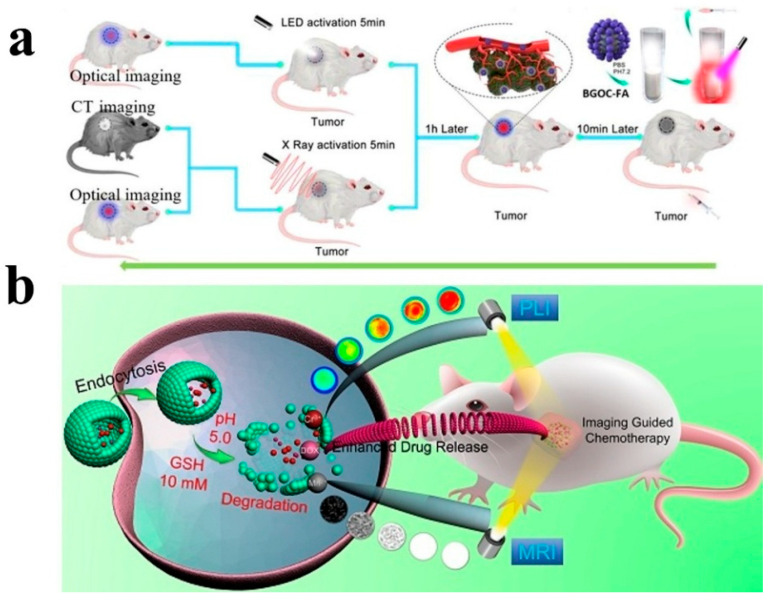
(**a**) PL/CT dual-modal imaging of BGOC nanoparticles injected into mice [179]. Copyright 2021 *Chemical Engineering Journal*. (**b**) Tumor targeted therapy guided by PLNPs MRI/NIR-PL dual-modality imaging [180]. Copyright 2021 *Theranostics*.

In 2021, Qu et al. [179] synthesized new Bi_2_Ga_4_O_9_: Cr (BGOC) PLNPs while combining optical imaging based on PLNPs with CT imaging to form dual-modality imaging (Figure 21a). It was found that the surface of the BGOC nanoparticles was modified by specific groups, which had high specificity and biocompatibility. Cr^3+^ ions were used as luminescent centers, and Bi^3+^ ions were used as contrast agents for CT imaging, which promoted PL and CT imaging in mice. At the same time, under the excitation of X-ray, the nanoprobe based on BGOC particles can effectively penetrate deep tissues and achieve long-term and high-sensitivity imaging of tumors in vivo. This probe also provides a promising imaging mode for high-sensitivity diagnosis and long-term monitoring of diseases. In the same year, Jiao et al. [180] developed Mn-doped hollow mesoporous silica shell structure, multi-functional NIR PLNPs, and guided in vivo tumor-targeted enhanced therapy through MRI/NIR-PL dual-modal imaging in response to the microenvironment in vivo (Figure 21b). The strategy has ultra-sensitive multi-mode diagnostic capabilities, enhanced anti-cancer effects, and efficient biodegradability, showing great transformation potential in precise cancer treatment.

**Figure 22:**
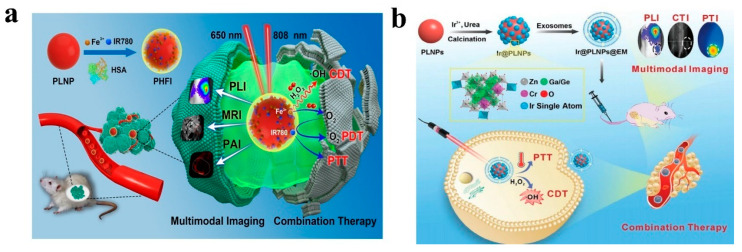
(**a**) Three-mode imaging and combined treatment of cancer sites in mice based on PLNPs [181]. Copyright 2020 *ACS Applied Materials & Interfaces*. (**b**) Nanozymes combined with single-atom Ir-coated persistent luminescent nanoparticles for multimodal imaging-guided combined tumor therapy [182]. Copyright 2024 *Advanced Healthcare Materials*.

In addition to bimodal imaging, in 2020, Wu et al. [181] displayed a multifunctional persistent luminescent nanoplatform for multimodality imaging and phototherapy of cancer (Figure 22a). The nanoplatform is composed of serum albumin, an IR780 probe, and near-infrared PLNPs modified by Fe^3+^ ions. The cancer site in mice is imaged by three modes of magnetic resonance and photoacoustic and persistent luminescence, which not only effectively kill tumor cells but also show a high longitudinal relaxation rate, obvious photoacoustic contrast signal, and persistent luminescence signal. This study offers a novel insight into cancer therapy and demonstrates significant application potential. In 2024, Ren et al. [182] combined Ir-coated Zn_1.2_Ga_1.6_Ge_0.2_O_4_: Cr PLNPs with near-infrared persistent luminescence properties with nanozymes and used near-infrared afterglow/photothermal/CT triple-peak imaging to guide photothermal-chemical kinetic combination therapy, successfully inhibiting tumor growth in vivo (Figure 22b). This study avoids the side effects of drug therapy, promotes the application of nanozymes and PLNPs, and develops a promising path for clinical diagnosis and treatment of cancer.

The above research shows that the integration of PLNPs and multimodal imaging technology has brought new development opportunities for biomedicine and has broad application prospects. In order to further promote the application of materials in imaging, future researchers need to develop a variety of new PLNPs to improve luminous efficiency, stability, and wavelength tunability so as to meet the needs of different imaging technologies. Concurrently, the design of multi-modal imaging probes is optimized, and a more diversified imaging system is formed through reasonable material combination and surface modification so as to realize the integration of imaging and treatment. In summary, the application of PLNPs in multimodal imaging will continue to expand and deepen, providing more accurate and effective tools for biomedical research and clinical diagnosis.

## 5. Application of PLNPs in Medical Treatment

In recent years, PLNPs have actively engaged in medical research. Relying on their unique advantages in bioimaging, they provide critical technical support for the spatial and temporal precision required in targeted therapy, giving rise to the cutting-edge diagnostic and therapeutic modality of “imaging-guided therapy”. Based on the medical treatment of PLNPs; the organic combination of PLNPs with functional substances such as chemotherapeutic drugs, photothermal conversion agents, photosensitizers, and immune drugs; as well as other functional materials such as photothermal nanomaterials and photodynamic nanomaterials, can not only exert the long-term biological imaging effect of PLNPs to achieve early and accurate diagnosis of diseases but also can simultaneously carry out various interventions such as photothermal therapy, photodynamic therapy, chemotherapy, or immunotherapy with the help of the therapeutic characteristics of the loaded functional materials. It is expected to break through the limitation of the separation of diagnosis and treatment and bring a new technical path and idea to the integration of the diagnosis and treatment of diseases. Table 4 compares the advantages and disadvantages of the four methods of nano-persistent luminescent materials for tumor treatment, which is helpful for the rational selection in practical applications and promotes the innovative development of personalized treatment schemes in the era of precision medicine.

### 5.1. Photodynamic Therapy

Photodynamic therapy (PDT) is a photochemical process that has been widely used in highly selective and non-invasive treatment of a variety of cancer lesions and other diseases. The principle of PDT is that photosensitizers are activated under light irradiation at a specific wavelength to produce high reactive oxygen species (ROS) such as singlet oxygen (^1^O_2_) with a strong oxidizing ability, which can oxidize intracellular biological macromolecules and destroy cell structure and function, thus directly killing tumor cells and achieving the purpose of treatment. PDT uses non-toxic photosensitizers and light, and its drug resistance is negligible. It has the characteristics of non-invasiveness and low side effects. However, traditional PDT requires a light source to continuously excite the photosensitizer, which inevitably causes damage to normal tissues. The excitation source band is mainly located in the range of visible light to near-infrared light. Limited by the depth of tissue penetration and scattering problems, it is not conducive to the effective treatment of deep tumors. The rapid development of nano-persistent luminescent materials provides a new possibility to solve this problem. Specifically, PLNPs do not require continuous excitation, and the ultra-long afterglow lifetime can store energy during a single irradiation. The energy is slowly transferred to the photosensitizer after the excitation stops to produce continuous ROS, achieving repeated charging in the body, minimizing the light damage caused by continuous irradiation, and improving the therapeutic performance of PDT in deep tissue.

**Figure 23:**
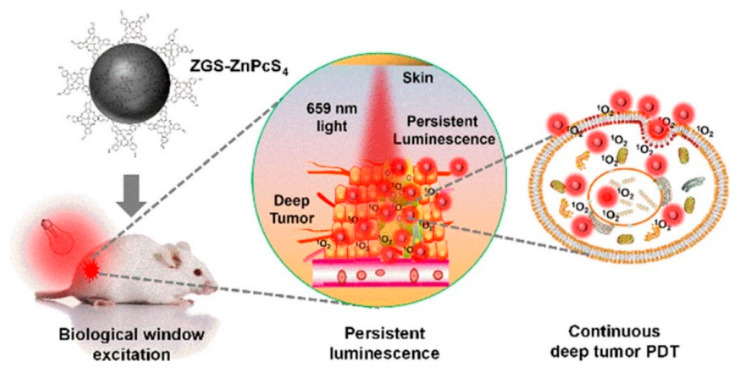
Functionalized ZGS nanoparticles for PDT treatment of deep tissue tumors [184]. Copyright 2020 *ACS Applied Bio Materials*.

In 2020, Liu et al. [183] constructed a persistent luminescent nanoparticle ZGGO: Cr, Bi @ mSiO_2_-ZnPc with deep red afterglow emission in the NIR I region for in-situ imaging-guided PDT. After 10 min of 635 nm red light irradiation, the inhibitory factor of cancer growth in mice was as high as 80%. This work shows the potential of afterglow nanomaterials in precision medicine. During the same year, Zhang et al. [184] doped Sn^4+^ into the ZnGa_2_O_4_ matrix and successfully synthesized NIR persistent luminescence Zn_1.3_Ga_1.4_Sn_0.3_O_4_: Cr^3+^ nanoparticles with a small particle size and excellent optical properties by the combustion method. The luminescence intensity surpassed 50 times that of the undoped counterpart. Subsequently, it was functionalized with ZnPcS_4_. Under irradiation of a 659 nm LED lamp, continuous and efficient tumor PDT treatment at a depth of 3 cm was achieved, as shown in Figure 23. This study largely solves the problem of low re-excitation efficiency after the decay of afterglow intensity of traditional PLNPs in deep tissue applications and provides a reference for further promoting the application of PLNPs in the biomedical field.

Although PLNPs excited by visible light have satisfactory biocompatibility and are easy to use for in vivo PDT treatment, their charging efficiency under visible light remains inadequate. In 2021, Wang et al. [185] engineered a set of X-ray-driven wavelength-tunable SrLaXO_4_: Bi^3+^ (X = Al, Ga, In) PLNPs, whose emission wavelength covers the region from UV to visible light, matching a g-C_3_N_4_ photosensitizer, suitable for deep tissue PDT therapy under X-ray irradiation. This design paves the way for new X-ray-activated persistent luminescent materials and shows broad prospects in PDT treatment of deep tumors.

**Figure 24:**
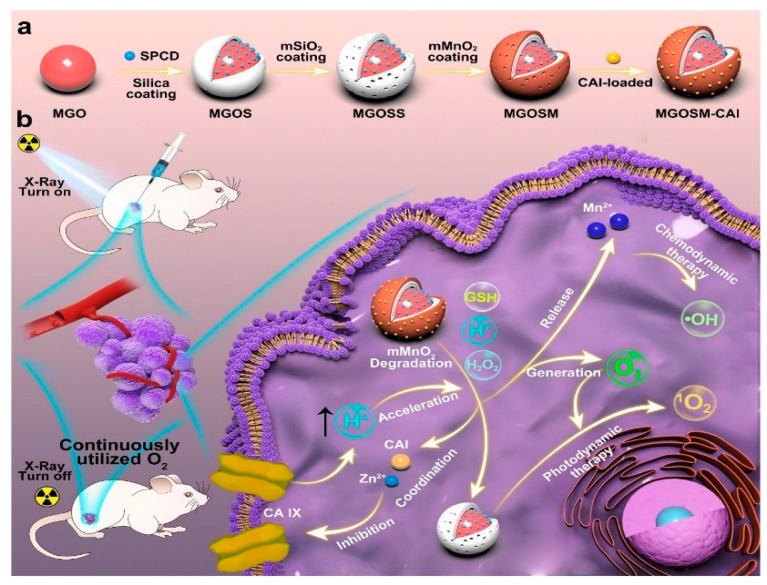
Functionalized persistent luminescent nanoparticles MGOSM-CAI for continuous PDT treatment of hypoxic tumors. (**a**) Schematic diagram of MGOSM-CAI preparation and (**b**) the mechanism for the effect of MGOSM-CAI on persistent hypoxic tumor treatment [186]. Copyright 2022 *Chemical Engineering Journal*.

In 2022, Shi et al. [186] loaded mesoporous MnO_2_, photosensitizer silicon phthalocyanine dihydroxide (SPCD), and a carbonic anhydrase inhibitor (CAI) onto MgGeO_3_: Mn^2+^, Yb^3+^ (MGO) PLNPs and developed an intelligent nanoplatform that can continuously use O_2_ for deep tissue tumor treatment, as shown in Figure 24. Given that mesoporous MnO_2_ accumulates in tumor cells, it can disassemble the high levels of H_2_O_2_ within the tumor microenvironment. This not only effectively mitigates tumor hypoxia but also triggers the release of CAI. Consequently, it could regulate cellular metabolism, remodel tumor microenvironment acidosis, and further accelerate MnO_2_ degradation and O_2_ generation. At the same time, X-ray irradiation of PLNPs to activate photosensitizers can consume O_2_, produce cytotoxic ^1^O_2_, and enhance PDT therapy. In addition, the released Mn^2+^ can also react with H_2_O_2_ to produce hydroxyl radicals that damage tumor cells. After the X-ray excitation is stopped, the whole process lasts for a long time to achieve continuous and improved treatment of hypoxic tumors. The present study introduces an innovative approach for addressing hypoxic tumors, demonstrating high efficacy in suppressing tumor progression and offering a robust strategy for managing deep-tissue hypoxic tumors. Apart from excitation with X-rays, in 2021, Su et al. [187] utilized Cerenkov radiation (CR)-emitting radionuclides as an internal excitation source to trigger photosensitizers for photodynamic therapy of deep-tissue tumors. In this experiment, ^131^I-labeled photosensitizer ZnPcC_4_ and ZnGa_2_O_4_: Cr^3+^ nanoparticles were functionalized PLNPs to construct a ^131^I-ZGCs-ZnPcC_4_ nanoplatform, which showed excellent tumor inhibition and less damage to surrounding normal tissues. The integration of continuous photodynamic therapy and radiotherapy has paved a novel avenue for addressing deep-seated tumors.

**Figure 25:**
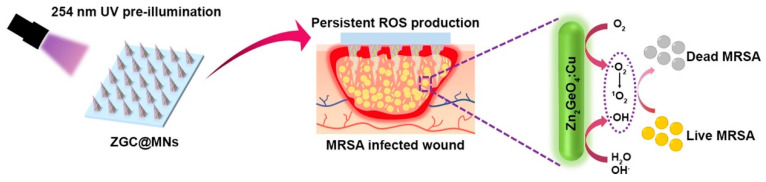
ZGC @ MN-based PDT for MRSA-infected wounds in vivo [188]. Copyright 2022 *ACS Applied Materials & Interfaces*.

In addition to the above research, PDT treatment has also become a promising method for fighting bacterial infections. In 2022, Yan et al. [188] reported a continuous luminescent nanorod Zn_2_GeO_4_: Cu^2+^ (ZGC) under 254 nm UV excitation as a penetrating biomembrane microneedle (MN) for the treatment of skin wound infections caused by methicillin-resistant Staphylococcus aureus (MRSA), as shown in Figure 25. In this work, ZGC nano-microneedles showed a continuous photocatalytic effect, producing ROS for up to 48 h at the infected wound, which not only promoted the dispersion of therapeutic drugs but also effectively inactivated bacteria, reduced inflammation, and accelerated wound healing. This innovative technology for removing MRSA bacterial infection does not involve in situ stimulation and does not require specific oxygen conditions, showing excellent antibacterial activity and biocompatibility, providing a new solution for reducing inflammation in vivo and promoting wound healing.

PLNPs have a good therapeutic effect and clinical application potential in PDT treatment. By rationally designing and preparing PLNPs, optimizing their combination with photosensitizers and energy transfer efficiency, the therapeutic effect can be further improved. In the future, it is necessary to further study the mechanism of action in treatment, solve the problems of biocompatibility and targeting of materials, and promote the PDT treatment technology mediated by PLNPs from laboratory research to clinical application, so as to bring good news to more patients.

### 5.2. Photothermal Therapy

Photothermal therapy (PTT) is a new type of treatment. The principle is that a photothermal conversion agent converts the absorbed light energy into heat energy under the irradiation of specific wavelength light, which increases the local temperature of tumor tissue and destroys the structure of cancer cells so as to achieve the purpose of treating cancer. Compared with traditional surgical treatment and radiotherapy, PTT has the advantages of being non-invasive to minimally invasive and has fewer side effects, strong repeatability, high selectivity, and targeting. Although there is a strong interest in PTT treatment, the method is still in its early stages and faces many challenges. The long afterglow luminescent material does not require real-time external excitation and shows obvious advantages with its unique luminescent properties. It avoids the inconvenience of continuous irradiation of the light source in traditional photothermal therapy and the potential damage to normal tissues, which provides a new possibility for PTT treatment.

In 2020, Wang et al. [189] prepared Zn_1.25_Ga_1.5_Ge_0.25_O_4_: Cr^3+^, Yb^3+^, Er^3+^ (ZGGO) persistent luminescent nanoparticles as a tracking center and coated it on mesoporous silica to form ZGGO @ SiO_2_. The photothermal fluorescent dye IR825 and the chemotherapeutic drug irinotecan were successively loaded through the silica pores. After 808 nm near-infrared light irradiation, colorectal cancer tracking, diagnosis, and precise treatment were achieved. The results showed that this strategy not only prevented the growth of tumors but also prolonged the survival time of tumor-bearing mice. This combination of chemotherapy and PTT guided by continuous luminescence imaging overcomes the limitations of single drug treatment and produces additional synergistic effects, providing a new model for the application of PLNPs in PPT. In 2021, Liu et al. [190] used cetyltrimethylammonium bromide (CTAB) and phosphotungstic acid (PW_12_) molecules to self-assemble and surface-modify PLNPs and gold nanorods to construct a biocompatible PLNP-GNR composite nanoplatform. Under the excitation of a single wavelength of 635 nm light, automatic non-fluorescence bioimaging and temperature changes during PTT in vitro and in vivo were monitored. The nanoplatform shows that the PLNPs act as an optical probe for bioimaging and a ratiometric afterglow nanothermometer with a temperature sensitivity of 0.064 K^−1^ and a photothermal conversion efficiency of 37%. This study successfully solved the difficulty of accurately locating the tumor and monitoring the temperature change during PTT in deep tissues, showing the great potential of multifunctional nanoplatforms integrated with bioimaging, temperature sensing, and PTT in the treatment of deep-tissue cancer.

**Figure 26:**
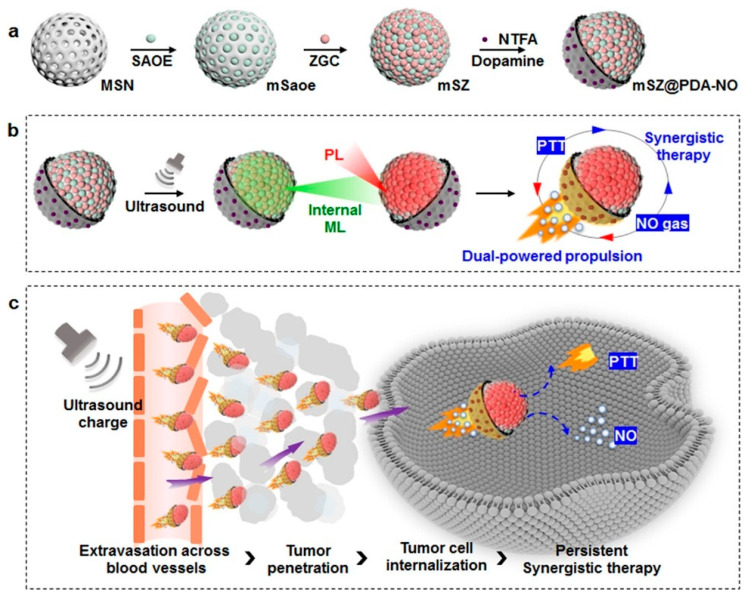
Preparation of mSZ @ PDA-NO multifunctional nanoparticles and its mechanism in PTT treatment. (**a**) Schematic illustration of the synthesis (**b**) Ultrasonication of nanoparticles induces ML green emission from SAOE nanodots, which excite ZGC nanodots to emit NIR PL (**c**) Nanoparticles achieve synergistic effects between PTT and NO release. [191]. Copyright 2023 *ACS Nano*.

In order to further solve the problem of tumor cell accessibility and intermittent light excitation in PTT, in 2023, Li et al. [191] developed an integrated platform for tumor diagnosis and treatment by continuously emitting nanoparticles to stimulate PTT while achieving thermophoresis-driven exercise and afterglow-triggered NO release. In the experiment, the researchers deposited mechano-luminescence nanodots SrAl_2_O_4_: Eu^2+^ (SAOE) and persistent luminescence nanodots ZnGa_2_O_4_: Cr^3+^ (ZGC) on mesoporous silicate, coated polydopamine (PDA) on the surface, and loaded an NO donor to prepare mSZ @ PDA-NO nanoparticles, forming a nanoplatform with multiple functions. Under the action of ultrasound, SAOE mediates mechanical luminescence, activates ZGC to emit persistent near-infrared long afterglow through energy transfer, provides a stable internal light source for deep tumors, and breaks through the traditional external light penetration depth limit (tissue penetration up to 10 cm). Moreover, near-infrared luminescence continuously excites PDA to trigger photothermal effects and trigger NO release. Its asymmetric structure can produce a dual driving mechanism of thermophoresis power and NO gas propulsion, giving nanoparticles autonomous movement ability, significantly improving tumor accumulation, interstitial infiltration, and cell uptake efficiency, as shown in Figure 26. Different from the commonly used intermittent NIR light irradiation, the system achieves long-term PTT treatment and intracellular NO sustained release by means of ultrasound-activated continuous luminescence and autonomous movement, avoiding the inconvenience of clinical operation caused by high-frequency light and reducing the risk of thermal damage to normal tissues. In addition, in vivo experiments have confirmed that this strategy can significantly inhibit tumor growth and prolong the survival time of mice without obvious systemic toxicity, providing an efficient and safe new strategy for deep tumor treatment, highlighting the unique application value of PLNPs in breaking through the key bottleneck of PTT.

### 5.3. Drug Delivery and Chemotherapy

Chemotherapy is one of the most commonly used cancer treatment methods in clinical medicine. Although it can interfere with the division of cancer cells and kill cancer cells, it has the disadvantages of poor targeting, large side effects, and strong drug resistance, which can easily damage normal tissues. Therefore, many nanoparticle-based drug delivery systems have been developed to effectively improve the accuracy and efficacy of chemotherapy by encapsulating drugs, targeted enrichment, and intelligent response release, providing a key path to break through the bottleneck of traditional chemotherapy. In this context, nano-persistent phosphorescent materials have realized long-term non-invasive imaging monitoring of the drug delivery process by virtue of the characteristics of continuous luminescence after excitation and constructed a multi-functional platform integrating targeted delivery, sustained drug release, and real-time imaging so as to promote the development of chemotherapy to the precision medical mode of diagnosis and treatment integration.

**Figure 27:**
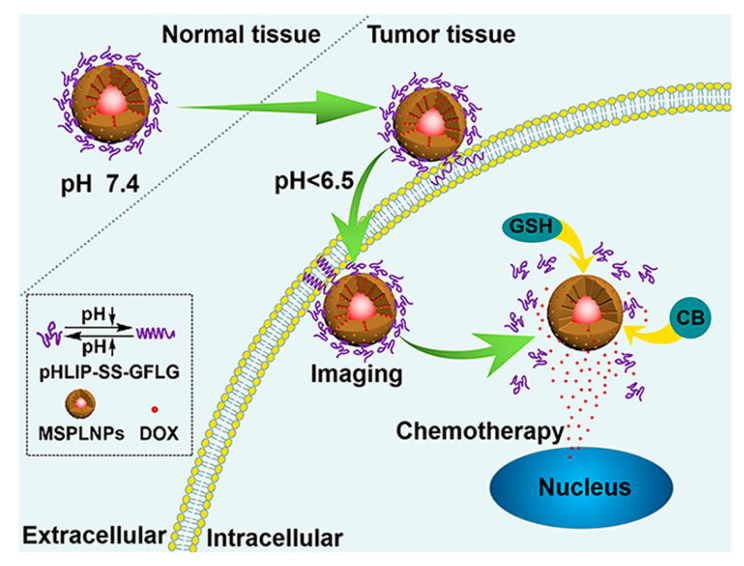
pH-driven targeted and CB/GSH dual-responsive drug release nanoprobes for continuous luminescence imaging and chemotherapy of tumors [193]. Copyright 2019 *Analytical Chemistry*.

Mesoporous silica is a typical drug delivery carrier with macroporous structure, high specific surface area, good biocompatibility, and low toxicity. In 2019, Fu et al. [192] used ZnGa_2_O_4_: Cr^3+^, Sn^4+^ (ZGCS) PLNPs as a signal source for repeatable luminescence imaging. Mesoporous silica (MS) was coated on the surface of ZGCS and loaded with the chemotherapeutic drug paclitaxel (Ptx). Hyaluronic acid (HA) was used as a targeting part to form a tumor-targeting, release-controllable multifunctional drug delivery nanoplatform for imaging-guided cancer chemotherapy. Experiments show that the nanoplatform can generate persistent luminescence under repeated excitation of 550 nm red light and achieve repeated luminescence imaging of cells or tissues. Meanwhile, the loading capacity of the platform to Ptx can reach 0.187 mg mg^−1^, which can specifically deliver drugs to target cells, enhance cytotoxicity to cancer cells, improve chemotherapy efficiency, and reduce side effects on normal cells. This study is expected to provide a promising drug delivery nanoplatform for imaging-guided cancer chemotherapy. In 2020, Yan et al. [193] developed a nanoprobe with pH-driven targeting and cathepsin B/glutathione dual-responsive drug release function for continuous luminescence imaging and chemotherapy of tumors. The probe is based on the core-shell structure of near-infrared PLNPs coated with mesoporous silica. The low-pH insertion peptide (pHLIP) is coupled to its surface through the GFLG peptide and disulfide bond as a connecting bridge, and the chemotherapeutic drug doxorubicin (DOX) is loaded in the pores. In the acidic cell microenvironment (pH < 6.5), the functionalized nanoprobe showed a higher tumor cell uptake rate than normal physiological conditions (pH=7.4) and a clear near-infrared persistent photoluminescence feature, which can enrich at the tumor site, achieving visual tumor-specific imaging unperturbed by autofluorescence. Additionally, a dual-responsive system containing cathepsin B and glutathione successfully triggered the release of doxorubicin loaded in mesoporous channels, effectively killing tumor cells and inhibiting tumor growth, as shown in Figure 27. Such nanoprobes for targeted tumor imaging and dual-stimuli-driven controlled drug release demonstrate substantial potential in theranostic applications, providing unique insights into the design of multifunctional nanoprobes.

Beyond mesoporous silica serving as a drug carrier, in 2023, Yan reported a persistent luminescent nanoparticle system encapsulated in a mesoporous polyacrylic acid (PAA)/calcium phosphate (CaP) shell (denoted as PLNPs@PAA/CaP). This system is designed to boost bioimaging quality and drug delivery effectiveness while mitigating bacterial infection-induced complications in chemotherapy [194]. Owing to shell-mediated defect passivation on the PLNP surface and intercomponent energy transfer, the encapsulation by a PAA/CaP shell effectively lengthens the afterglow persistence and triples the luminescence intensity. Simultaneously, the negatively charged mesoporous architecture of the PAA/CaP shell enables efficient loading of the cationic antibiotic doxycycline hydrochloride. In the acidic microenvironment of bacterial infection, the shell degrades rapidly and releases the drug, thereby effectively killing the bacteria at the infected site. Boasting exceptional long-lasting luminescence characteristics, superior biocompatibility, and rapid responsiveness to drug release, this nanoplatform is anticipated to emerge as a highly promising tool in clinical therapy, significantly enhancing chemotherapy efficacy while mitigating adverse effects.

### 5.4. Immunotherapy

Immunotherapy is one of the most promising anti-cancer treatment methods, which has the characteristics of safety, specificity, persistence, and wide indications. The principle is based on the regulation of the immune system by activating or enhancing the body’s own immune system to identify and attack tumor cells to achieve the purpose of treating cancer. In numerous cancer types, immunotherapy has substantially boosted both survival rates and quality of life in patients. Over the recent decades, researchers have formulated a range of approaches to modulate tumor-specific immune responses, encompassing the induction of immunogenic cell death (ICD) and the blockade of immune checkpoints. In particular, the expression of damage-associated molecular pattern molecules (DAMPs) could be upregulated in tumor cells treated with ICD. DAMPs are exposed to the immune system for a long time, which can initiate strong immune stimulation and induce long-lasting anti-cancer immunity. Oxidative stress based on reactive oxygen species (ROS) is one of the efficient ways to initiate ICD. PLNPs are capable of sustained ROS generation upon a single illumination, which imparts it with marked potential to drive continuous ICD in the context of cancer immunotherapy.

**Figure 28:**
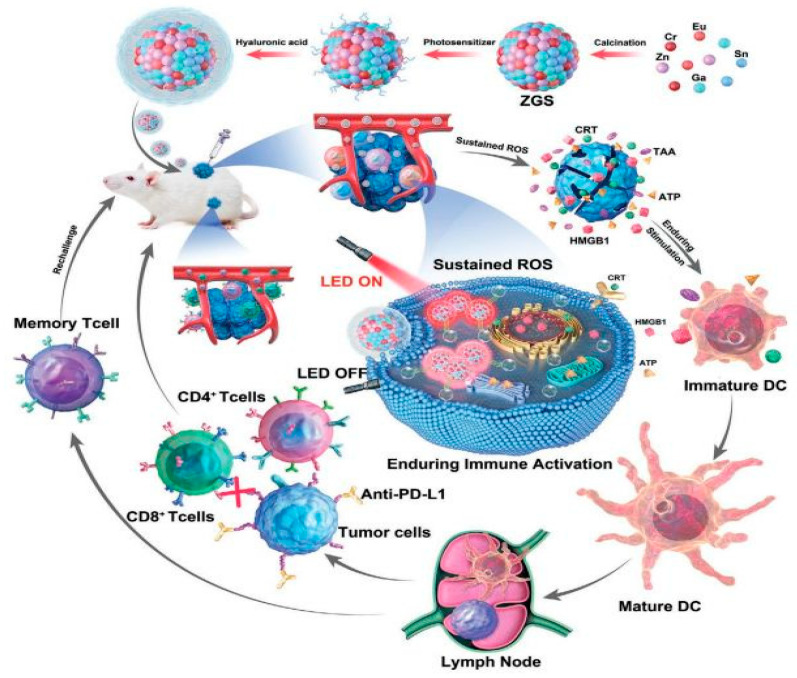
Immunostimulant ZGS-Si-Pc@HA based on PLNPs for long-lasting tumor-specific immunotherapy [195]. Copyright 2021 *Advanced Functional Materials*.

In 2021, Zhang and his colleagues modified the surface of Zn_3_Ga_2_Sn_2_O_10_: Cr^3+^, Eu^2+^ (ZGS: Cr, Eu) PLNPs with photosensitizer Si-Pc and further encapsulated them with hyaluronic acid (HA) to construct a novel targeted nano-immunostimulant for continuous immunotherapy (ZGS-Si-Pc@HA) [195]. Upon irradiation with 659 nm light, ZGS-Si-Pc@HA could perpetually trigger ROS-mediated ICD, thereby eliciting a sustained tumor-specific immune response (Figure 28). In vivo experiments showed that ZGS-Si-Pc@HA effectively alleviated immune tolerance and promoted tumor infiltration of cytotoxic T lymphocytes after intratumoral injection.

PLNPs can not only trigger ICD through ROS but also serve as an immune adjuvant to enhance the ability of immune cells to recognize and kill tumor cells. When PLNPs enter the body, they are taken up by antigen-presenting cells such as macrophages and dendritic cells, which can transmit tumor antigen signals to T cells, activate the immune response of T cells, and promote their killing effect on tumor cells. In addition, PLNPs can also release cytokines and chemokines, attract more immune cells to accumulate in the tumor site, increase the infiltration of immune cells in the tumor tissue, and further enhance the killing effect on tumor cells. In 2021, Sun et al. [196] employed a “solid-to-gel” strategy to incorporate persistent luminescent materials and the immunoadjuvant R837 into alginate Ca^2+^ hydrogels for rechargeable photodynamic immunotherapy of tumors. The designed PLM-R837-ALG hydrogel exhibited characteristics of persistent luminescence, good biocompatibility, and injectability, enabling easy injection into tumors as an internal light source to effectively activate photosensitizers and induce sustained photodynamic therapy (PDT) effects. The loaded R837 immunoadjuvant significantly enhanced the immunogenicity of damage-associated molecular patterns (DAMPs) released by tumor cells after immunogenic cell death (ICD) therapy, thereby triggering a robust systemic anti-tumor immune response, inhibiting in vivo tumors, and achieving sustained immunotherapy. This persistent photodynamic immunotherapy expands the possibilities for effective tumor treatment and provides a novel paradigm for cancer therapy.

**Figure 29:**
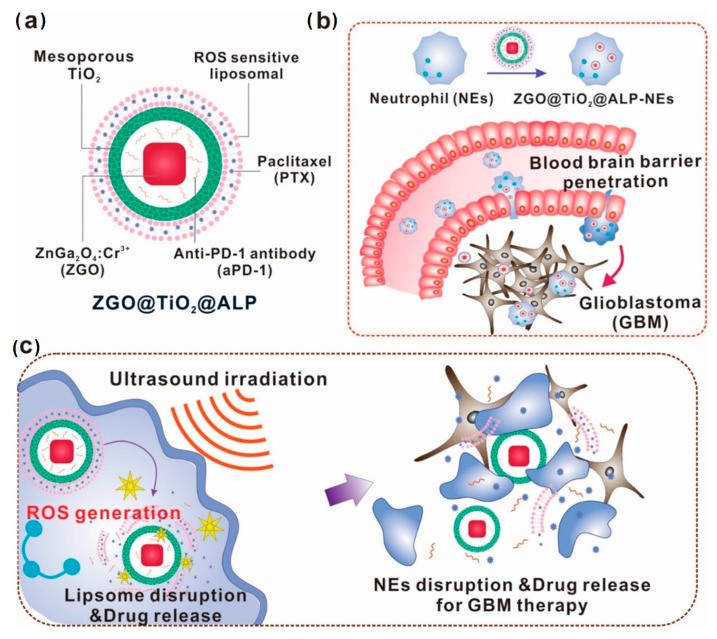
Hollow titanium dioxide-coated persistent luminescent nanosensitizer for ultrasound-amplified chemotherapy and immunotherapy of glioblastoma. (**a**) Composition of ZGO@TiO_2_@APL. (**b**) ZGO@TiO_2_@APL was loaded by neutrophils and attracted by GBM inflammation. (**c**) Ultrasound triggered drug release from ZGO@TiO_2_@APL-NEs for GBM immunotherapy [197]. Copyright 2021 *Advanced Science*.

In addition to the aforementioned studies, PLNPs can also cooperate with immune checkpoint inhibitors to inhibit the growth of bilateral tumors and trigger immune memory effects. In 2021, Li et al. [197] developed a neutrophil delivery nanosensitizer for ultrasound-enhanced chemotherapy and immunotherapy of glioblastoma. The nanosensitizer is composed of hollow titanium dioxide (TiO_2_) shell-coated persistent luminescent nanoparticles ZnGa_2_O_4_: Cr^3+^ (ZGO), internally loaded with the immune checkpoint inhibitor anti-PD-1 antibody to alleviate the immunosuppression of glioblastoma and externally loaded with paclitaxel (PTX) liposomes to achieve chemical inhibition of glioblastoma, as shown in Figure 29. After intravenous injection, under ultrasound irradiation, the sensitizer continuously produces ROS, causing the liposome layer to be destroyed, releasing PTX and anti-PD-1 antibodies to kill tumor cells and induce local inflammation, thereby attracting more nanoparticles to migrate to the tumor site to enhance the continuous treatment effect. The results showed that this immunotherapy strategy increased the survival rate of mice from 0% to 40%, provided long-term immune monitoring ability for tumor recurrence, and provided a new method for precise treatment of glioblastoma and other cancers.

PLNPs show a multi-dimensional mechanism of action and significant application potential in immunotherapy. Whether they are used as a medium to induce ICD, an immune adjuvant, or in combination with immune checkpoint inhibitors, they provide new ideas and methods for cancer immunotherapy. However, the related research is still in the exploratory stage. In the future, it is necessary to further study their mechanism of action, optimize the material properties, and carry out more preclinical and clinical trials to promote the wide application of PLNPs in the field of immunotherapy.

## 6. Conclusions and Prospect

This paper systematically reviews the latest research progress on PLNPs in biological detection and imaging and their medical treatment applications in recent years. With the unique advantages of ultra-long afterglow life, no real-time excitation, no tissue background signal, biological autofluorescence signal interference, and high SNR, this material has shown great application potential in the biomedical field and has become a hot topic in biomedical research. However, PLNPs are still in the developmental phase, with pending issues in material performance tuning, biosafety appraisal, application extension, and clinical translation that warrant further investigation. To push forward research in this field, the subsequent sections will tackle these challenges by centering on five key aspects: the synthesis of PLNPs, afterglow wavelength modulation, biosafety evaluation, expanded biomedical applications, and clinical translation strategies.

(1) Synthesis of materials: Despite the fact that numerous synthetic methods for PLNPs have been reported, none of these methods can be deemed optimal. They cannot simultaneously meet the requirements for the morphological characteristics, homogeneity, and afterglow properties of PLNPs but only enhance one or several of these attributes. Therefore, we need to further develop more advanced and controllable synthesis strategies. On the one hand, the luminescent and biological properties of materials can be enhanced through doping with specific ions or surface modification during synthesis. On the other hand, the limitations of existing synthesis methods can be overcome by optimizing processes, simplifying procedures, integrating multiple synthetic strategies, and incorporating machine learning, thus facilitating the fabrication of high-performance PLNPs.
(2) Excitation and emission wavelengths of the afterglow: The excitation light source is one of the most important factors affecting the biomedical application of PLNPs. Specifically, UV excitation yields an insufficient afterglow duration to support biological applications, necessitating repeated excitation cycles. Consequently, the study of excitation light will focus on NIR, X-rays, and radionuclides. Meanwhile, the emission wavelengths of most PLNPs are concentrated in the NIR-I region, whereas research on NIR-II/III PLNPs remains relatively scarce. It is therefore imperative to strengthen investigations into NIR-II/III PLNPs, as this could enhance tissue penetration depth and imaging performance. In addition, the development of corresponding commercially available imaging instruments is expected to overcome the technical bottlenecks in NIR-II/III region research, thereby further expanding their biomedical applications.
(3) Biological safety of materials: Most existing PLNPs are doped with various transition metal ions or lanthanide ions, both of which may induce potential harmful side effects due to their long-term retention and deposition in the body. Thus, they need to be studied toxicologically. For example, through genomics, proteomics, and metabolomics approaches, we can further examine the long-term toxicity, possible immunotoxicity, metabolic pathways, and biostructural distribution of PLNPs in vivo, which can lead to in-depth biosafety assessment.
(4) Expansion of biomedical applications: Although there has been a significant breakthrough in the application of PLNPs, their full potential remains to be exploited. Notably, PLNPs exhibit relatively limited application scenarios in biodetection, highlighting the necessity of developing multi-wavelength emissive PLNPs to facilitate the assay of diverse substances. Beyond focusing on the diagnostic and therapeutic dimensions of tumors, we hope to develop PLNPs oriented to special applications, such as deep tissue imaging in vivo, transfer tracking of nerve or cell signals, and diagnosis of special diseases. Furthermore, smart imaging-guided therapy systems, as the emerging and urgent trend, may lead to a more intelligent response to PLNPs, which is of great significance for the development of PLNPs.
(5) Clinical transformation: The research on PLNPs at this stage mostly stays in the laboratory stage, and achieving a leap from basic research to clinical trials is a major challenge in this field. In the future, it is necessary to strengthen multi-party cooperation, improve the pre-clinical research data of materials, establish a standardized production process and quality control system, and formulate evaluation criteria in line with clinical application norms so as to promote the early clinical transformation of PLNPs and benefit patients.

## Figures and Tables

**Table 1:** Characteristics of different matrix LPLMs.

Matrix	Typical Material	Afterglow Colors	Emission Peak (nm)	Persistence	Feature	Ref.
Sulfide	CaS: Eu, Dy ZnS: Cu, Co SrS: Eu, Dy	Red Green Orange	650 525 607	5 h 3 h ˃60 min	Various emission color, Poor stability, short persistency	[39,40,41]
Aluminate	SrAl_2_O_4_: Eu, Dy CaAl_2_O_4_: Eu, Nd Sr_4_Al_14_O_25_: Eu, Dy	Green Blue Cyan	520 450 490	60 h 20 h 20 h	High light, long lasting time, low water resistance and high cost	[42,43,44]
Silicate	Sr_2_MgSi_2_O_7_: Eu, Dy Ca_2_MgSi_2_O_7_: Eu, Dy Ca_2_ZnSi_2_O_7_: Eu, Dy	Blue Green Yellow	470 535 580	10 h 12 h 12 h	Cheap, good stability and water resistance, slightly low luminescence time and efficiency	[45,46,47]
Gallium and Germanate	ZnGa_2_O_4_: Cr Zn_3_Ga_2_Ge_2_O_10_: Cr La_3_Ga_5_GeO_14_: Cr CaZnGe_2_O_6_: Mn, Bi	Red-near infrared	600–800 650–1000 700–1300 648	12 h 360 h 8 h 3 h	Good chemical and thermal stability, strong penetrability, widely used in biomedicine	[48,49,50,51]
Others	Ca_4_Ti_3_O_10_: Pr Zn_2_SnO_4_: Cr Sr_3_B_2_O_6_: Sm Sr_2_P_2_O_7_: Eu, Y	Red NIR Orange Violet	612 800 598 420	3 h ˃2 min ˃2 h ˃8 h	Diversification, complementarity of different matrix materials, wider application	[52,53,54,55]

**Table 3:** Comparison of several luminescent materials for biological detection.

Material	Excitation	Emission	Quantum Yield	Lifetime	Biological Characteristics	SNR	Refs.
Organic dyes	UV-Vis-NIR	Vis-NIR-II	Medium, dependence structure	Nanosecond	Low toxicity, poor stability, low penetration ability	Low	[122,123,124]
Quantum dots	UV-Vis-NIR	UV-Vis-NIR-III	High, strong absorption	Nanosecond	High toxicity, good stability, middle biocompatibility	Middle	[125,126,127]
Upconversion nanoparticles	NIR	UV-Vis-NIR-II	Low, dependence on doped ions	Nanosecond	Low toxicity, continuous external excitation	High	[128,129,130]
Long persistent luminescent nanoparticles	X-rays-UV-Vis-NIR	UV-Vis-NIR-III	Medium	Up to tens of hours	Low toxicity, no continuous external excitation	Ultra-high	[131,132,133]

**Table 4:** Comparison of different approaches using PLNPs in cancer therapy.

Therapies	Principle	Advantage	Disadvantage
Photodynamic Therapy	Photosensitizers generate reactive oxygen species (such as singlet oxygen) under light to destroy tumor cells	High specificity, non-invasive, little injury, low toxicity, repeatable treatment	Dependent on oxygen and external light source, limited penetrability, restricted photosensitizer selection
Photothermal Therapy	Conversion of absorbed light energy into heat by photothermal conversion agents to generate high temperature and destroy tumor cells	Non-invasiveness, precise heating, no drug resistance	Limited penetration depth, high-temperature-induced damage to surrounding normal tissues
Chemotherapy	Nanomaterials loaded with chemotherapeutic drugs for passive or active targeting delivery to tumor sites	Systemic therapy, combination with other therapies	High side effects, prone to drug resistance, uncontrolled drug release
Immunotherapy	Specific activation of the immune system for recognition and attack of tumor cells	Safety, persistence, long-term immune memory, potential curative effect	Slow onset, immune escape, high interindividual variability

## Data Availability

No new data were created or analyzed in this study. Data sharing is not applicable to this article.

## References

[1] Liao S., Zhou M., Wang Y., Lu C., Yin B., Zhang Y., Liu H., Yin X., Song G. (2023). Emerging biomedical imaging-based companion diagnostics for precision medicine. iScience.

[2] Pinto-Coelho L. (2023). How Artificial Intelligence Is Shaping Medical Imaging Technology: A Survey of Innovations and Applications. Bioengineering.

[3] Yang X., Huang K., Yang D., Zhao W., Zhou X. (2024). Biomedical Big Data Technologies, Applications, and Challenges for Precision Medicine: A Review. Glob. Chall..

[4] Han X., Xu K., Taratula O., Farsad K. (2019). Applications of nanoparticles in biomedical imaging. Nanoscale.

[5] Keall P.J., Brighi C., Glide-Hurst C., Liney G., Liu P.Z.Y., Lydiard S., Paganelli C., Trang P., Shan S., Tree A.C. (2022). Integrated MRI-guided radiotherapy—Opportunities and challenges. Nat. Rev. Clin. Oncol..

[6] Lother D., Robert M., Elwood E., Smith S., Tunariu N., Johnston S.R., Parton M., Bhaludin B., Millard T., Downey K. (2023). Imaging in metastatic breast cancer, CT, PET/CT, MRI, WB-DWI, CCA: Review and new perspectives. Cancer Imaging.

[7] Wang S., Ren W.X., Hou J.-T., Won M., An J., Chen X., Shu J., Kim J.S. (2021). Fluorescence imaging of pathophysiological microenvironments. Chem. Soc. Rev..

[8] Jiang Y., Pu K. (2021). Molecular Probes for Autofluorescence-Free Optical Imaging. Chem. Rev..

[9] Jaiswal S., Das S., Kundu S., Rawal I., Anand P., Patra A. (2022). Progress and perspectives: Fluorescent to long-lived emissive multifunctional probes for intracellular sensing and imaging. J. Mater. Chem. C.

[10] Dou W.-T., Han H.-H., Sedgwick A.C., Zhu G.-B., Zang Y., Yang X.-R., Yoon J., James T.D., Li J., He X.-P. (2022). Fluorescent probes for the detection of disease-associated biomarkers. Sci. Bull..

[11] Ojha A., Ojha N.K. (2021). Excitation light-induced phototoxicity during fluorescence imaging. J. Biosci..

[12] Dai J., Zhang X. (2023). Chemical Regulation of Fluorescence Lifetime. Chem. Biomed. Imaging.

[13] Kasprzycka W., Szumigraj W., Wachulak P., Trafny E.A. (2024). New approaches for low phototoxicity imaging of living cells and tissues. BioEssays.

[14] Pei P., Chen Y., Sun C., Fan Y., Yang Y., Liu X., Lu L., Zhao M., Zhang H., Zhao D. (2021). X-ray-activated persistent luminescence nanomaterials for NIR-II imaging. Nat. Nanotechnol..

[15] Liang L., Chen J., Shao K., Qin X., Pan Z., Liu X. (2023). Controlling persistent luminescence in nanocrystalline phosphors. Nat. Mater..

[16] Xu J., Tanabe S. (2019). Persistent luminescence instead of phosphorescence: History, mechanism, and perspective. J. Lumin..

[17] Yang S., Dai W., Zheng W., Wang J. (2023). Non-UV-activated persistent luminescence phosphors for sustained bioimaging and phototherapy. Coord. Chem. Rev..

[18] Huang K., Le N., Wang J.S., Huang L., Zeng L., Xu W.C., Li Z., Li Y., Han G. (2022). Designing Next Generation of Persistent Luminescence: Recent Advances in Uniform Persistent Luminescence Nanoparticles. Adv. Mater..

[19] Li Y., Gecevicius M., Qiu J. (2016). Long persistent phosphors-from fundamentals to applications. Chem. Soc. Rev..

[20] Zhou Z., Li Y., Peng M. (2020). Near-infrared persistent phosphors: Synthesis, design, and applications. Chem. Eng. J..

[21] Vaidyanathan S. (2023). Recent progress on lanthanide-based long persistent phosphors: An overview. J. Mater. Chem. C.

[22] Yu N., Li Y., Li Z., Han G. (2018). The “bottom-up” synthesis and applications of persistent luminescence nanoparticles. Sci. China Chem..

[23] Huang Z., Chen B., Ren B., Tu D., Wang Z., Wang C., Zheng Y., Li X., Wang D., Ren Z. (2023). Smart Mechanoluminescent Phosphors: A Review of Strontium-Aluminate-Based Materials, Properties, and Their Advanced Application Technologies. Adv. Sci..

[24] Wu S., Li Y., Ding W., Xu L., Ma Y., Zhang L. (2020). Recent Advances of Persistent Luminescence Nanoparticles in Bioapplications. Nano-Micro Lett..

[25] Chan M.-H., Chang Y.-C. (2024). Recent advances in near-infrared I/II persistent luminescent nanoparticles for biosensing and bioimaging in cancer analysis. Anal. Bioanal. Chem..

[26] Sun S.-K., Wang H.-F., Yan X.-P. (2018). Engineering Persistent Luminescence Nanoparticles for Biological Applications: From Biosensing/Bioimaging to Theranostics. Acc. Chem. Res..

[27] Liu J., Lecuyer T., Seguin J., Mignet N., Scherman D., Viana B., Richard C. (2019). Imaging and therapeutic applications of persistent luminescence nanomaterials. Adv. Drug Deliv. Rev..

[28] Liu N., Chen X., Sun X., Sun X., Shi J. (2021). Persistent luminescence nanoparticles for cancer theranostics application. J. Nanobiotechnol..

[29] Lastusaari M., Laamanen T., Malkamaki M., Eskola K.O., Kotlov A., Carlson S., Welter E., Brito H.F., Bettinelli M., Jungner H. (2012). The Bologna Stone: history’s first persistent luminescent material. Eur. J. Mineral..

[30] Hölsä J. (2009). Persistent Luminescence Beats the Afterglow: 400 Years of Persistent Luminescence. Electrochem. Soc. Interface.

[31] Smet P.F., Moreels I., Hens Z., Poelman D. (2010). Luminescence in Sulfides: A Rich History and a Bright Future. Materials.

[32] Matsuzawa T., Aoki Y., Takeuchi N., Murayama Y. (1996). A New Long Phosphorescent Phosphor with High Brightness, SrAl_2_O_4_: Eu^2+^, Dy^3+^. J. Electrochem. Soc..

[33] le Masne de Chermont Q., Chaneac C., Seguin J., Pelle F., Maitrejean S., Jolivet J.-P., Gourier D., Bessodes M., Scherman D. (2007). Nanoprobes with near-infrared persistent luminescence for in vivo imaging. Proc. Natl. Acad. Sci. USA.

[34] Maldiney T., Kaikkonen M.U., Seguin J., de Chermont Q.L.M., Bessodes M., Airenne K.J., Yla-Herttuala S., Scherman D., Richard C. (2012). In Vitro Targeting of Avidin-Expressing Glioma Cells with Biotinylated Persistent Luminescence Nanoparticles. Bioconjugate Chem..

[35] Li L., Li T., Hu Y., Cai C., Li Y., Zhang X., Liang B., Yang Y., Qiu J. (2022). Mechanism of the trivalent lanthanides’ persistent luminescence in wide bandgap materials. Light Sci. Appl..

[36] Di Giorgio E., Campolucci M., Alberti S., Locardi F. (2024). From Bulk to Nano: The Effect on the Persistent Luminescence. Cryst. Growth Des..

[37] Yang L., Gai S., Ding H., Yang D., Feng L., Yang P. (2023). Recent Progress in Inorganic Afterglow Materials: Mechanisms, Persistent Luminescent Properties, Modulating Methods, and Bioimaging Applications. Adv. Opt. Mater..

[38] Kasim L., Li B., Abdukayum A. (2024). Preparation and Application of Inorganic Persistent Luminescent Composite Materials. Prog. Chem..

[39] Rodriguez Burbano D.C., Sharma S.K., Dorenbos P., Viana B., Capobianco J.A. (2015). Persistent and Photostimulated Red Emission in CaS: Eu^2+^, Dy^3+^ Nanophosphors. Adv. Opt. Mater..

[40] Ma L., Chen W. (2011). Enhancement of Afterglow in ZnS: Cu, Co Water-Soluble Nanoparticles by Aging. J. Phys. Chem. C.

[41] Kong M., Fang M., Tan X., Liu M., Shang G.L., Fei G.T., Zhang L. (2018). The investigation on the mechanism of the increased decay time in red SrS: Eu^2+^, Dy^3+^ phosphor. Mater. Chem. Phys..

[42] Estefania Rojas-Hernandez R., Rubio-Marcos F., Angel Rodriguez M., Francisco Fernandez J. (2018). Long lasting phosphors: SrAl_2_O_4_: Eu, Dy as the most studied material. Renew. Sustain. Energy Rev..

[43] Qu B., Zhang B., Wang L., Zhou R., Zeng X.C. (2015). Mechanistic Study of the Persistent Luminescence of CaAl_2_O_4_: Eu, Nd. Chem. Mater..

[44] Demirci S., Gultekin S., Akalin S.A., Oter O., Ertekin K., Celik E. (2015). Synthesis and spectral characterization of Sr_4_Al_14_O_25_: Eu^2+^/Dy^3+^ blue-green phosphorous powders by sol-gel method. Mater. Sci. Semicond. Process..

[45] Pan L., Liu S., Zhang X., Oderinde O., Yao F., Fu G. (2018). Optimization method for blue Sr_2_MgSi_2_O_7_: Eu^2+^, Dy^3+^ phosphors produced by microwave synthesis route. J. Alloys Compd..

[46] Sahu I.P., Bisen D.P., Tamrakar R.K., Shrivastava R. (2016). Enhancement of the photoluminescence and long afterglow properties of Ca_2_MgSi_2_O_7_: Eu^2+^ phosphor by Dy^3+^ co-doping. Res. Chem. Intermed..

[47] Jiang L., Xiao S., Yang X., Zhang X., Liu X., Zhou B., Jin X. (2013). Preparation and luminescence properties of yellow long-lasting phosphor Ca_2_ZnSi_2_O_7_: Eu^2+^, Dy^3+^. Mater. Sci. Eng. B-Adv. Funct. Solid-State Mater..

[48] Srivastava B.B., Gupta S.K., Mohan S., Mao Y. (2021). Molten-Salt-Assisted Annealing for Making Colloidal ZnGa_2_O_4_: Cr Nanocrystals with High Persistent Luminescence. Chemistry.

[49] Pan Z., Lu Y.-Y., Liu F. (2012). Sunlight-activated long-persistent luminescence in the near-infrared from Cr^3+^-doped zinc gallogermanates. Nat. Mater..

[50] Yan W., Liu F., Lu Y.-Y., Wang X.-J., Yin M., Pan Z. (2010). Near infrared long-persistent phosphorescence in La_3_Ga_5_GeO_14_: Cr^3+^ phosphor. Opt. Express.

[51] Ye K., Yang X., Xiao S. (2021). Improving red afterglow properties of CaZnGe_2_O_6_: Mn^2+^ by co-doping Bi^3+^. Optik.

[52] Wang B., Li X., Chen Y., Chen Y., Zhou J., Zeng Q. (2018). Long persistent and photo-stimulated luminescence in Pr^3+^-doped layered perovskite phosphor for optical data storage. J. Am. Ceram. Soc..

[53] Taktak O., Souissi H., Kammoun S. (2020). Optical properties of the phosphors Zn_2_SnO_4_: Cr^3+^ with near-infrared long-persistence phosphorescence for bio-imaging applications. J. Lumin..

[54] Pekgozlu I. (2019). A Novel Reddish Orange Luminescent Material Sr_3_B_2_O_6_: Sm^3+^. J. Appl. Spectrosc..

[55] Ju G., Hu Y., Chen L., Wang X., Mu Z. (2013). The influence of auxiliary codopants on persistent phosphor Sr_2_P_2_O_7_: Eu^2+^, R^3+^ (R = Y, La, Ce, Gd, Tb and Lu). Mater. Res. Bull..

[56] Xu S., Chen R., Zheng C., Huang W. (2016). Excited State Modulation for Organic Afterglow: Materials and Applications. Adv. Mater..

[57] Nidhankar A.D., Goudappagouda, Wakchaure V.C., Babu S.S. (2021). Efficient metal-free organic room temperature phosphors. Chem. Sci..

[58] Shen H., Liao S., Li Z., Wang Y., Huan S., Zhang X.B., Song G. (2023). Organic Afterglow Nanoparticles in Bioapplications. Chemistry.

[59] Fan Y., Li Q., Li Z. (2023). Afterglow bio-applications by utilizing triplet excited states of organic materials. Sci. China Chem..

[60] Zhao Z., Du R., Feng X., Wang Z., Wang T., Xie Z., Yuan H., Tan Y., Ou H. (2025). Regulating Triplet Excitons of Organic Luminophores for Promoted Bioimaging. Curr. Med. Chem..

[61] Gong Y., Tan Y., Li H., Zhang Y., Yuan W., Zhang Y., Sun J., Tang B.Z. (2013). Crystallization-induced phosphorescence of benzils at room temperature. Sci. China-Chem..

[62] An Z., Zheng C., Tao Y., Chen R., Shi H., Chen T., Wang Z., Li H., Deng R., Liu X. (2015). Stabilizing triplet excited states for ultralong organic phosphorescence. Nat. Mater..

[63] Xie Y., Ge Y., Peng Q., Li C., Li Q., Li Z. (2017). How the Molecular Packing Affects the Room Temperature Phosphorescence in Pure Organic Compounds: Ingenious Molecular Design, Detailed Crystal Analysis, and Rational Theoretical Calculations. Adv. Mater..

[64] Yang Z., Ubba E., Huang Q., Mao Z., Li W., Chen J., Zhao J., Zhang Y., Chi Z. (2020). Enabling dynamic ultralong organic phosphorescence in molecular crystals through the synergy between intramolecular and intermolecular interactions. J. Mater. Chem. C.

[65] Xiao G., Fang X., Ma Y.-J., Yan D. (2022). Multi-Mode and Dynamic Persistent Luminescence from Metal Cytosine Halides through Balancing Excited-State Proton Transfer. Adv. Sci..

[66] Wang J., Gu X., Ma H., Peng Q., Huang X., Zheng X., Sung S.H.P., Shan G., Lam J.W.Y., Shuai Z. (2018). A facile strategy for realizing room temperature phosphorescence and single molecule white light emission. Nat. Commun..

[67] Wang W., Zhang Y., Jin W.J. (2020). Halogen bonding in room-temperature phosphorescent materials. Coord. Chem. Rev..

[68] Shi H., Yao W., Ye W., Ma H., Huang W., An Z. (2022). Ultralong Organic Phosphorescence: From Material Design to Applications. Acc. Chem. Res..

[69] Zhu J., Zhao L., An W., Miao Q. (2025). Recent advances and design strategies for organic afterglow agents to enhance autofluorescence-free imaging performance. Chem. Soc. Rev..

[70] Peng Q., Ma H., Shuai Z. (2021). Theory of Long-Lived Room-Temperature Phosphorescence in Organic Aggregates. Acc. Chem. Res..

[71] Singh M., Liu K., Qu S., Ma H., Shi H., An Z., Huang W. (2021). Recent Advances of Cocrystals with Room Temperature Phosphorescence. Adv. Opt. Mater..

[72] Chen H., Yao X., Ma X., Tian H. (2016). Amorphous, Efficient, Room-Temperature Phosphorescent Metal-Free Polymers and Their Applications as Encryption Ink. Adv. Opt. Mater..

[73] Cai S., Ma H., Shi H., Wang H., Wang X., Xiao L., Ye W., Huang K., Cao X., Gan N. (2019). Enabling long-lived organic room temperature phosphorescence in polymers by subunit interlocking. Nat. Commun..

[74] Li J.-A., Zhang L., Wu C., Huang Z., Li S., Zhang H., Yang Q., Mao Z., Luo S., Liu C. (2023). Switchable and Highly Robust Ultralong Room-Temperature Phosphorescence from Polymer-Based Transparent Films with Three-Dimensional Covalent Networks for Erasable Light Printing. Angew. Chem.-Int. Ed..

[75] Li H., Xue X., Cao Y., Cheng H., Luo A., Guo N., Li H., Xie G., Tao Y., Chen R. (2023). Achieving Stimuli-Responsive Amorphous Organic Afterglow in Single-Component Copolymer through Self-Doping. J. Am. Chem. Soc..

[76] Lu Y., Chen X., Suh Y.D., Liu X., Huang W. (2023). Rigidity-Mediated Afterglow Tuning of Small Molecules in Polymer Matrix through Photoinitiated Solvent-Free Copolymerization. Adv. Opt. Mater..

[77] Zhang Z.-C., Gu Z.-G., Zhang J. (2024). Host–Guest Metal–Organic Frameworks-Based Long-Afterglow Luminescence Materials. Molecules.

[78] Zhou B., Yan D. (2023). Long Persistent Luminescence from Metal-Organic Compounds: State of the Art. Adv. Funct. Mater..

[79] Liu J., Yang L.Y., Luo F. (2021). A New Zn-Triazole MOF Showing Very Long-Lived Luminescence up to 3 S. J. Solid State Chem..

[80] Yu X., Ryadun A.A., Pavlov D.I., Guselnikova T.Y., Potapov A.S., Fedin V.P. (2024). Ln-MOF-Based Hydrogel Films with Tunable Luminescence and Afterglow Behavior for Visual Detection of Ofloxacin and Anti-Counterfeiting Applications. Adv. Mater..

[81] Yuan J., Dong J., Lei S., Hu W. (2021). Long afterglow MOFs: A frontier study on synthesis and applications. Mater. Chem. Front..

[82] Qin X., Wang J., Yuan Q. (2020). Synthesis and Biomedical Applications of Lanthanides-Doped Persistent Luminescence Phosphors With NIR Emissions. Front. Chem..

[83] Thejo Kalyani N., Jain A., Dhoble S.J. (2022). Persistent phosphors for luminous paints: A review. Luminescence.

[84] Mushtaq U., Ayoub I., Yagoub M.Y.A., Som S., Swart H.C., Kumar V. (2023). Structural and photoluminescent properties of Gd^3+^ doped barium aluminate phosphor. Phys. B-Condens. Matter.

[85] Liu P., Zhang Y., Li B., Han L., Xu Y. (2022). Trap depth engineering in MgGa_2_O_4_: Bi^3+^ for muticolor dynamic anti-counterfeiting, encryption and optical temperature sensing applications. Chem. Eng. J..

[86] Wang X., Chen Y., Liu F., Pan Z. (2020). Solar-blind ultraviolet-C persistent luminescence phosphors. Nat. Commun..

[87] Abdukayum A., Chen J.-T., Zhao Q., Yan X.-P. (2013). Functional Near Infrared-Emitting Cr^3+^/Pr^3+^ Co-Doped Zinc Gallogermanate Persistent Luminescent Nanoparticles with Superlong Afterglow for in Vivo Targeted Bioimaging. J. Am. Chem. Soc..

[88] Meroni D., Porati L., Demartin F., Poelman D. (2017). Sol–Gel Synthesis of CaTiO_3_: Pr^3+^ Red Phosphors: Tailoring the Synthetic Parameters for Luminescent and Afterglow Applications. ACS Omega.

[89] Mori K., Onoda H., Toyama T., Kojima Y. (2019). Synthesis and fluorescence studies of Eu^3+^-doped SrAl_12_O_19_ phosphor. Optik.

[90] Zhao B., Zhu Q., Sun X., Li J.-G. (2021). Co-doping Zn^2+^/Sn^4+^ in ZnGa_2_O_4_: Cr^3+^ for dynamic near-infrared luminescence and advanced anti-counterfeiting. Ceram. Int..

[91] Jin M., Li F., Xiahou J., Zhu L., Zhu Q., Li J.-G. (2023). A new persistent luminescence phosphor of ZnGa_2_O_4_: Ni^2+^ for the second near-infrared transparency window. J. Alloys Compd..

[92] Ayoub I., Mushtaq U., Yagoub M.Y.A., Som S., Swart H.C., Kumar V. (2023). Structural and optical characteristics of green-emitting BaGd_2_ZnO_5_: Tb^3+^ phosphor for LED applications. Phys. B-Condens. Matter.

[93] Pan L., Delaey M., Wang Y., Poelman D. (2024). Structural and optical properties of Cr ion-doped near-infrared long persistent luminescence silicogermanate phosphors with broad emission bands. J. Alloys Compd..

[94] Li J.-L., Shi J.-P., Wang C.-C., Li P.-H., Yu Z.-F., Zhang H.-W. (2017). Five-nanometer ZnSn_2_O_4_: Cr, Eu ultra-small nanoparticles as new near infrared-emitting persistent luminescent nanoprobes for cellular and deep tissue imaging at 800 nm. Nanoscale.

[95] Hu S., Li Z., Luo Q., Ma Q., Chen N., Fu L., Wang J., Yang R., Yuan Q. (2019). Facile Synthesis of Luminous Nanoparticles with Tunable Size and Long-Lived Luminescence for Lifetime-Based Biosensing. Cryst. Growth Des..

[96] Fu L., Wang J., Chen N., Ma Q., Lu D., Yuan Q. (2020). Enhancement of long-lived luminescence in nanophosphors by surface defect passivation. Chem. Commun..

[97] Singh S., Tanwar V., Simantilleke A.P., Kumar H., Singh D. (2020). Structural and spectroscopic properties of CaMgSi_2_O_6_: RE^3+^ (Eu^3+^ and Tb^3+^) nanophosphors under UV-illumination. Optik.

[98] Wu M., Wang Y., Wang Y., Wu S., Shen Y. (2022). Effect of Citric Acid Amount in the Synthesis of LiGa_5_O_8_: Cr^3+^ Nano-Phosphor. Russ. J. Phys. Chem. A.

[99] Wu M.Y., Wang Y., Shen Y., Li F.F., Wang J., Liu Y., Peng C. (2021). Preparation of Zn_3_Ga_2_Ge_2_O_10_: Cr^3+^, Al^3+^ nanometer phosphors via sol-gel method. Dig. J. Nanomater. Biostruct..

[100] Zou R., Huang J., Shi J., Huang L., Zhang X., Wong K.-L., Zhang H., Jin D., Wang J., Su Q. (2017). Silica shell-assisted synthetic route for mono-disperse persistent nanophosphors with enhanced in vivo recharged near-infrared persistent luminescence. Nano Res..

[101] Wang J., Li J., Yu J., Zhang H., Zhang B. (2018). Large Hollow Cavity Luminous Nanoparticles with Near-Infrared Persistent Luminescence and Tunable Sizes for Tumor Afterglow Imaging and Chemo-/Photodynamic Therapies. ACS Nano.

[102] Feng Y., Liu R., Zhang L., Li Z., Su Y., Lv Y. (2019). Raspberry-Like Mesoporous Zn_1.07_Ga_2.34_Si_0.98_O_6.56_: Cr_0.01_ Nanocarriers for Enhanced Near-Infrared Afterglow Imaging and Combined Cancer Chemotherapy. ACS Appl. Mater. Interfaces.

[103] Tan R., Wu J., Wang C., Zhao Z., Zhang X., Zhong C., Tang Z., Zheng R., Du B., He Y. (2025). The develop of persistent luminescence nanoparticles with excellent performances in cancer targeted bioimaging and killing: A review. J. Nanobiotechnol..

[104] Wang J., Ma Q., Hu X.-X., Liu H., Zheng W., Chen X., Yuan Q., Tan W. (2017). Autofluorescence-Free Targeted Tumor Imaging Based on Luminous Nanoparticles with Composition-Dependent Size and Persistent Luminescence. ACS Nano.

[105] Li Z., Zhang Y., Wu X., Huang L., Li D., Fan W., Han G. (2015). Direct Aqueous-Phase Synthesis of Sub-10 nm “Luminous Pearls” with Enhanced in Vivo Renewable Near-Infrared Persistent Luminescence. J. Am. Chem. Soc..

[106] Shi J., Sun X., Zheng S., Li J., Fu X., Zhang H. (2018). A new near-infrared persistent luminescence nanoparticle as a multifunctional nanoplatform for multimodal imaging and cancer therapy. Biomaterials.

[107] Wang J., Li Q., Zhao H., Yue W., Zhang K., Jiang X., Li K. (2022). Facile and Controllable Synthesis of the Renal-Clearable “Luminous Pearls” for in Vivo Afterglow/Magnetic Resonance Imaging. ACS Nano.

[108] Vitola V., Millers D., Bite I., Smits K., Spustaka A. (2019). Recent progress in understanding the persistent luminescence in SrAl_2_O_4_: Eu, Dy. Mater. Sci. Technol..

[109] Sun X., Song L., Liu N., Shi J., Zhang Y. (2021). Chromium-Doped Zinc Gallate Near-Infrared Persistent Luminescence Nanoparticles in Autofluorescence-Free Biosensing and Bioimaging: A Review. ACS Appl. Nano Mater..

[110] Yang X., Waterhouse G.I.N., Lu S., Yu J. (2023). Recent advances in the design of afterglow materials: Mechanisms, structural regulation strategies and applications. Chem. Soc. Rev..

[111] Abbruscato V. (1971). Optical and Electrical Properties of SrAl_2_O_4_: Eu^2+^. J. Electrochem. Soc..

[112] Qi Z., Shi C., Liu M., Zhou D., Luo X., Zhang J., Xie Y. (2004). The valence of rare earth ions in R_2_MgSi_2_O_7_: Eu, Dy (R = Ca, Sr) long-afterglow phosphors. Phys. Status Solidi (A).

[113] Clabau F., Rocquefelte X., Jobic S., Deniard P., Whangbo M.-H., Garcia A., Mercier T.L. (2005). Mechanism of Phosphorescence Appropriate for the Long-Lasting Phosphors Eu^2+^-Doped SrAl_2_O_4_ with Codopants Dy^3+^ and B^3+^. Chem. Mater..

[114] Aitasalo T., Holsa J., Jungner H., Lastusaari M., Niittykoski J. (2006). Thermoluminescence study of persistent luminescence materials: Eu^2+^- and R^3+^-doped calcium aluminates, CaAl_2_O_4_: Eu^2+^, R^3+^. J. Phys. Chem. B.

[115] Dorenbos P., Bos A.J.J., Poolton N.R.J. (2011). Electron transfer processes in double lanthanide activated YPO_4_. Opt. Mater..

[116] Dorenbos P., Shalapska T., Stryganyuk G., Gektin A., Voloshinovskii A. (2011). Spectroscopy and energy level location of the trivalent lanthanides in LiYP_4_O_12_. J. Lumin..

[117] Lecointre A., Bessiere A., Bos A.J.J., Dorenbos P., Viana B., Jacquart S. (2011). Designing a Red Persistent Luminescence Phosphor: The Example of YPO_4_: Pr^3+^, Ln^3+^ (Ln = Nd, Er, Ho, Dy). J. Phys. Chem. C.

[118] Denis G., Deniard P., Gautron E., Clabau F., Garcia A., Jobic S. (2008). Structure and white luminescence of Eu-activated (Ba,Sr)_13-x_Al_22-2x_Si_10+2x_O_66_ materials. Inorg. Chem..

[119] Liu Y., Wang Z., Miao K., Zhang X., Li W., Zhao P., Sun P., Zheng T., Zhang X., Chen C. (2022). Research progress on near-infrared long persistent phosphor materials in biomedical applications. Nanoscale Adv..

[120] Zhao X., Chen L.-J., Zhao K.-C., Liu Y.-S., Liu J.-L., Yan X.-P. (2019). Autofluorescence-free chemo/biosensing in complex matrixes based on persistent luminescence nanoparticles. Trac-Trends Anal. Chem..

[121] Huang P., Tu D., Zheng W., Zhou S., Chen Z., Chen X. (2015). Inorganic lanthanide nanoprobes for background-free luminescent bioassays. Sci. China-Mater..

[122] Resch-Genger U., Grabolle M., Cavaliere-Jaricot S., Nitschke R., Nann T. (2008). Quantum dots versus organic dyes as fluorescent labels. Nat. Methods.

[123] Bradburne C.E., Delehanty J.B., Gemmill K.B., Mei B.C., Mattoussi H., Susumu K., Blanco-Canosa J.B., Dawson P.E., Medintz I.L. (2013). Cytotoxicity of Quantum Dots Used for In Vitro Cellular Labeling: Role of QD Surface Ligand, Delivery Modality, Cell Type, and Direct Comparison to Organic Fluorophores. Bioconjug. Chem..

[124] Abdel-Mottaleb M.M.A., Beduneau A., Pellequer Y., Lamprecht A. (2015). Stability of fluorescent labels in PLGA polymeric nanoparticles: Quantum dots versus organic dyes. Int. J. Pharm..

[125] Algar W.R., Massey M., Rees K., Higgins R., Krause K.D., Darwish G.H., Peveler W.J., Xiao Z., Tsai H.-Y., Gupta R. (2021). Photoluminescent Nanoparticles for Chemical and Biological Analysis and Imaging. Chem. Rev..

[126] Chung S., Revia R.A., Zhang M. (2021). Graphene Quantum Dots and Their Applications in Bioimaging, Biosensing, and Therapy. Adv. Mater..

[127] Das S., Mondal S., Ghosh D. (2024). Carbon quantum dots in bioimaging and biomedicines. Front. Bioeng. Biotechnol..

[128] Wang L., Draz M.S., Wang W., Liao G., Xu Y. (2015). The Quality of In Vivo Upconversion Fluorescence Signals Inside Different Anatomic Structures. J. Biomed. Nanotechnol..

[129] Li K., Hong E., Wang B., Wang Z., Zhang L., Hu R., Wang B. (2019). Advances in the application of upconversion nanoparticles for detecting and treating cancers. Photodiagn. Photodyn. Ther..

[130] Li H., Sheng W., Haruna S.A., Hassan M.M., Chen Q. (2023). Recent advances in rare earth ion-doped upconversion nanomaterials: From design to their applications in food safety analysis. Compr. Rev. Food Sci. Food Saf..

[131] Wang J., Ma Q., Wang Y., Shen H., Yuan Q. (2017). Recent progress in biomedical applications of persistent luminescence nanoparticles. Nanoscale.

[132] Wei Y., Gong C., Zhao M., Zhang L., Yang S., Li P., Ding Z., Yuan Q., Yang Y. (2022). Recent progress in synthesis of lanthanide-based persistent luminescence nanoparticles. J. Rare Earths.

[133] Sun M., Chen M., Wang J. (2024). Perspective and Prospects on Persistent Luminescent Nanoparticles for Biological Imaging and Tumor Therapy. Curr. Med. Chem..

[134] Lecuyer T., Teston E., Ramirez-Garcia G., Maldiney T., Viana B., Seguin J., Mignet N., Scherman D., Richard C. (2016). Chemically engineered persistent luminescence nanoprobes for bioimaging. Theranostics.

[135] Ferlay J., Colombet M., Soerjomataram I., Mathers C., Parkin D.M., Pineros M., Znaor A., Bray F. (2019). Estimating the global cancer incidence and mortality in 2018: GLOBOCAN sources and methods. Int. J. Cancer.

[136] Li M., Zhao J., Chu H., Mi Y., Zhou Z., Di Z., Zhao M., Li L. (2019). Light-Activated Nanoprobes for Biosensing and Imaging. Adv. Mater..

[137] Fu L., Qian Y., Zhou J., Zheng L., Wang Y. (2020). Fluorescence-based quantitative platform for ultrasensitive food allergen detection: From immunoassays to DNA sensors. Compr. Rev. Food Sci. Food Saf..

[138] Zhang H., Ma X., Yu L., He S., Zhu R., Meng Z. (2025). Constructing “off-on” luminescence biosensor based on fluorescence resonance energy transfer for autofluorescence-free detection of uric acid and glucose. Inorg. Chem. Commun..

[139] Dang Q., Jiang Y., Wang J., Wang J., Zhang Q., Zhang M., Luo S., Xie Y., Pu K., Li Q. (2020). Room-Temperature Phosphorescence Resonance Energy Transfer for Construction of Near-Infrared Afterglow Imaging Agents. Adv. Mater..

[140] Hanif H., Ali M.J., Susheela A.T., Khan I.W., Luna-Cuadros M.A., Khan M.M., Lau D.T.-Y. (2022). Update on the applications and limitations of alpha-fetoprotein for hepatocellular carcinoma. World J. Gastroenterol..

[141] Wu B.-Y., Wang H.-F., Chen J.-T., Yan X.-P. (2011). Fluorescence Resonance Energy Transfer Inhibition Assay for α-Fetoprotein Excreted during Cancer Cell Growth Using Functionalized Persistent Luminescence Nanoparticles. J. Am. Chem. Soc..

[142] Feng F., Chen X., Li G., Liang S., Hong Z., Wang H.-F. (2018). Afterglow Resonance Energy Transfer Inhibition for Fibroblast Activation Protein-α Assay. ACS Sens..

[143] Pan Z., Yang D., Lin J., Shao K., Shi S., Teng Y.-J., Liu H., She Y. (2022). Autofluorescence free detection of carcinoembryonic antigen in pleural effusion by persistent luminescence nanoparticle-based aptasensors. Anal. Chim. Acta.

[144] Zhao L., Chen J., Pang Y., Fu K., Shang Q., Wu H., Sun L., Lin Q., Chen H. (2022). Fibroblast activation protein-based theranostics in cancer research: A state-of-the-art review. Theranostics.

[145] Wu B.-Y., Yan X.-P. (2015). Bioconjugated persistent luminescence nanoparticles for Föster resonance energy transfer immunoassay of prostate specific antigen in serum and cell extracts without in situ excitation. Chem. Commun..

[146] Wang X., Wang Y., Chen S., Fu P., Lin Y., Ye S., Long Y., Gao G., Zheng J. (2022). A persistent luminescence resonance energy transfer-based molecular beacon probe for the highly sensitive detection of microRNA in biological samples. Biosens. Bioelectron..

[147] Feng Y., Zhang L., Liu R., Lv Y. (2019). Modulating near-infrared persistent luminescence of core-shell nanoplatform for imaging of glutathione in tumor mouse model. Biosens. Bioelectron..

[148] Li N., Li Y., Han Y., Pan W., Zhang T., Tang B. (2014). A Highly Selective and Instantaneous Nanoprobe for Detection and Imaging of Ascorbic Acid in Living Cells and in Vivo. Anal. Chem..

[149] Pavao M.L., Ferin R., Lima A., Baptista J. (2022). Cysteine and related aminothiols in cardiovascular disease, obesity and insulin resistance. Adv. Clin. Chem..

[150] Li J., Yang C., Wang W.-L., Yan X.-P. (2018). Functionalized gold and persistent luminescence nanoparticle-based ratiometric absorption and TR-FRET nanoplatform for high-throughput sequential detection of L-cysteine and insulin. Nanoscale.

[151] Iqbal H., Yang T., Li T., Zhang M., Ke H., Ding D., Deng Y., Chen H. (2021). Serum protein-based nanoparticles for cancer diagnosis and treatment. J. Control. Release.

[152] Liu Y., Wang Y., Jiang K., Sun S., Qian S., Wu Q., Lin H. (2020). A persistent luminescence-based label-free probe for the ultrasensitive detection of hemoglobin in human serum. Talanta.

[153] Channer B., Matt S.M., Nickoloff-Bybel E.A., Pappa V., Agarwal Y., Wickman J., Gaskill P.J. (2023). Dopamine, Immunity, and Disease. Pharmacol. Rev..

[154] Yao T., Dong G., Qian S., Cui Y., Chen X., Tan T., Li L. (2022). Persistent luminescence nanoparticles/hierarchical porous ZIF-8 nanohybrids for autoluminescence-free detection of dopamine. Sens. Actuators B-Chem..

[155] Zhang Y., Zhou J., Zhang X.-X., Wang W.-L., Yang C., Shi X., Feng Y.-W., Abdurahman R. (2022). NIR persistent luminescence nanoparticles based turn-on aptasensor for autofluorescence-free determination of 17β-estradiol in milk. Food Chem..

[156] Guo J.-X., Pan L.-M., Wang M.-C., Chen L.-J., Zhao X. (2023). Exogenous interference and autofluorescence-free ratiometric aptasensor for detection of OTA based on dual-colored persistent luminescence nanoparticles. Food Chem..

[157] Su B., Zhang Z., Sun Z., Tang Z., Xie X., Chen Q., Cao H., Yu X., Xu Y., Liu X. (2022). Fluonanobody-based nanosensor via fluorescence resonance energy transfer for ultrasensitive detection of ochratoxin A. J. Hazard. Mater..

[158] Zhao Q., Lu D., Zhang G., Zhang D., Shi X. (2021). Recent improvements in enzyme-linked immunosorbent assays based on nanomaterials. Talanta.

[159] Uniyal A., Srivastava G., Pal A., Taya S., Muduli A. (2023). Recent Advances in Optical Biosensors for Sensing Applications: A Review. Plasmonics.

[160] Liang L., Chen N., Jia Y., Ma Q., Wang J., Yuan Q., Tan W. (2019). Recent progress in engineering near-infrared persistent luminescence nanoprobes for time-resolved biosensing/bioimaging. Nano Res..

[161] Jia M., Zhang Z., Li J., Ma X., Chen L., Yang X. (2018). Molecular imprinting technology for microorganism analysis. TrAC Trends Anal. Chem..

[162] Zhang L., Lei J., Liu J., Ma F., Ju H. (2015). Persistent luminescence nanoprobe for biosensing and lifetime imaging of cell apoptosis via time-resolved fluorescence resonance energy transfer. Biomaterials.

[163] Chen S., Cai G., Gong X., Wang L., Cai C., Gong H. (2022). Non-autofluorescence Detection of H5N1 Virus Using Photochemical Aptamer Sensors Based on Persistent Luminescent Nanoparticles. ACS Appl. Mater. Interfaces.

[164] Liu H., Li Z., Shen R., Li Z., Yang Y., Yuan Q. (2021). Point-of-Care Pathogen Testing Using Photonic Crystals and Machine Vision for Diagnosis of Urinary Tract Infections. Nano Lett..

[165] Feng Y., Chen T., Rao Q., Xie X., Lv Y., Zhang L. (2022). Time-Resolved Persistent Luminescence Encoding for Multiplexed Severe Acute Respiratory Syndrome Coronavirus 2 Detection. Anal. Chem..

[166] Xue J., Li F., Liu F., Noh H.M., Lee B.R., Choi B.C., Park S.H., Jeong J.H., Du P. (2022). Designing ultra-highly efficient Mn^2+^-activated Zn_2_GeO_4_ green-emitting persistent phosphors toward versatile applications. Mater. Today Chem..

[167] Wu S., Li Y., Zhang R., Fan K., Ding W., Xu L., Zhang L. (2021). Persistent luminescence-polypyrrole nanocomposite for dual-modal imaging and photothermal therapy of mammary cancer. Talanta.

[168] Lin X., Zhang K., Li Y., Nan F., Li J., Zhang H., Deng W., Ding W., Li K., Jarhen N. (2024). High resolution osteoclast-targeted imaging-guided osteoporosis alleviation via persistent luminescence nanocomposite. Chem. Eng. J..

[169] Luo X., Shi J., Wang R., Cao L., Gao Y., Wang J., Hong M., Sun X., Zhang Y. (2024). Near-Infrared Persistent Luminescence Nanoprobe for Early Detection of Atherosclerotic Plaque. ACS Nano.

[170] Maldiney T., Bessiere A., Seguin J., Teston E., Sharma S.K., Viana B., Bos A.J.J., Dorenbos P., Bessodes M., Gourier D. (2014). The in vivo activation of persistent nanophosphors for optical imaging of vascularization, tumours and grafted cells. Nat. Mater..

[171] Zhong X., Wang X., Li J., Hu J., Cheng L., Yang X. (2021). ROS-based dynamic therapy synergy with modulating tumor cell-microenvironment mediated by inorganic nanomedicine. Coord. Chem. Rev..

[172] Wei Y., Wang J. (2024). X-ray/γ-ray/Ultrasound-Activated Persistent Luminescence Phosphors for Deep Tissue Bioimaging and Therapy. ACS Appl. Mater. Interfaces.

[173] Qiu X., Zhu X., Xu M., Yuan W., Feng W., Li F. (2017). Hybrid Nanoclusters for Near-Infrared to Near-Infrared Upconverted Persistent Luminescence Bioimaging. ACS Appl. Mater. Interfaces.

[174] Chen X., Li Y., Huang K., Huang L., Tian X., Dong H., Kang R., Hu Y., Nie J., Qiu J. (2021). Trap Energy Upconversion-Like Near-Infrared to Near-Infrared Light Rejuvenateable Persistent Luminescence. Adv. Mater..

[175] Hu Y., Li X., Wang X., Li Y., Li T., Kang H., Zhang H., Yang Y. (2020). Greatly enhanced persistent luminescence of YPO_4_: Sm^3+^ phosphors via Tb^3+^ incorporation for in vivo imaging. Opt. Express.

[176] Liu B.-M., Zou R., Lou S.-Q., Gao Y.-F., Ma L., Wong K.-L., Wang J. (2021). Low-dose X-ray-stimulated LaGaO_3_: Sb, Cr near-infrared persistent luminescence nanoparticles for deep-tissue and renewable in vivo bioimaging. Chem. Eng. J..

[177] Liu N., Shi J., Wang Q., Guo J., Hou Z., Su X., Zhang H., Sun X. (2020). In Vivo Repeatedly Activated Persistent Luminescence Nanoparticles by Radiopharmaceuticals for Long-Lasting Tumor Optical Imaging. Small.

[178] Lin Y., Hu J., Guo Y., Zou Q., Chen D., Xu K., Liang S., Yi X., Lu H., Wang S.-B. (2023). Enhanced persistent luminescence of MgGeO_3_: Yb^3+^ nanoparticles via substitution of Ge^4+^ by Ga^3+^ ions for biological applications. J. Lumin..

[179] Li J., Guo J., Li H., Qu J., Song J. (2021). Simultaneous realization of persistent luminescence and CT dual-mode imaging by X-ray recharged Bi_2_Ga_4_O_9_: Cr nanoprobes in depth-independent tumors. Chem. Eng. J..

[180] Zou R., Li J., Yang T., Zhang Y., Jiao J., Wong K.-L., Wang J. (2021). Biodegradable manganese engineered nanocapsules for tumor-sensitive near-infrared persistent luminescence/magnetic resonance imaging and simultaneous chemotherapy. Theranostics.

[181] Wu S., Qian Z., Li Y., Hu S., Ma Y., Wei S., Zhang L. (2020). Persistent Luminescence Nanoplatform with Fenton-like Catalytic Activity for Tumor Multimodal Imaging and Photoenhanced Combination Therapy. ACS Appl. Mater. Interfaces.

[182] Li Y., Wu S.-Q., Nan F., Deng W., Li K., Jarhen N., Zhou Y., Ma Q., Qu Y., Chen C. (2024). Single-Atom Iridium Nanozyme-Based Persistent Luminescence Nanoparticles for Multimodal Imaging-Guided Combination Tumor Therapy. Adv. Healthc. Mater..

[183] Yang J., Zhao Y., Meng Y., Zhu H., Yan D., Liu C., Xu C., Zhang H., Xu L., Li Y. (2020). Irradiation-free photodynamic therapy in vivo induced by enhanced deep red afterglow within NIR-I bio-window. Chem. Eng. J..

[184] Shi J., Sun X., Zheng S., Song L., Zhang F., Madl T., Zhang Y., Zhang H., Hong M. (2020). Tin-Doped Near-Infrared Persistent Luminescence Nanoparticles with Considerable Improvement of Biological Window Activation for Deep Tumor Photodynamic Therapy. ACS Appl. Bio Mater..

[185] Liu B.-M., Gan W.-J., Lou S.-Q., Zou R., Tang Q., Wang C.-X., Jiao J., Wang J. (2021). X-ray-activated, UVA persistent luminescent materials based on Bi-doped SrLaAlO_4_ for deep-Seated photodynamic activation. J. Appl. Phys..

[186] Lin P., Shi J., Ming L., Sheng Y., Song L., Hong M., Zhang Y. (2022). An intelligent persistent luminescence nanoplatform with high-efficiency O_2_ utilization for continuous hypoxic tumors treatment. Chem. Eng. J..

[187] Wang Q., Liu N., Hou Z., Shi J., Su X., Sun X. (2021). Radioiodinated Persistent Luminescence Nanoplatform for Radiation-Induced Photodynamic Therapy and Radiotherapy. Adv. Healthc. Mater..

[188] Gong J.-H., Chen L.-J., Zhao X., Yan X.-P. (2022). Persistent Production of Reactive Oxygen Species with Zn_2_GeO_4_: Cu Nanorod-Loaded Microneedles for Methicillin-Resistant Staphylococcus Aureus Infectious Wound Healing. ACS Appl. Mater. Interfaces.

[189] Wang Z.-H., Liu J.-M., Zhao N., Li C.-Y., Lv S.-W., Hu Y., Lv H., Wang D., Wang S. (2020). Cancer Cell Macrophage Membrane Camouflaged Persistent Luminescent Nanoparticles for Imaging-Guided Photothermal Therapy of Colorectal Cancer. ACS Appl. Nano Mater..

[190] Meng Y., Yang J., Jiang R., Wang S., Zheng L., Wang G., Tian X., Zhu H., Yan D., Liu C. (2021). Biocompatible PLNP-GNR composite nanoplatforms for monitoring deep-tissue photothermal therapy process. Appl. Surf. Sci..

[191] Zhang Z., Yan H., Cao W., Xie S., Ran P., Wei K., Li X. (2023). Ultrasound-Chargeable Persistent Luminescence Nanoparticles to Generate Self-Propelled Motion and Photothermal/NO Therapy for Synergistic Tumor Treatment. ACS Nano.

[192] Jiang W., Huang L., Mo F., Zhong Y., Xu L., Fu F. (2019). Persistent luminescent multifunctional drug delivery nano-platform based on nanomaterial ZnGa_2_O_4_: Cr^3+^, Sn^4+^ for imaging-guided cancer chemotherapy. J. Mater. Chem. B.

[193] Zhang H.-J., Zhao X., Chen L.-J., Yang C.-X., Yan X.-P. (2020). pH-Driven Targeting Nanoprobe with Dual-Responsive Drug Release for Persistent Luminescence Imaging and Chemotherapy of Tumor. Anal. Chem..

[194] Fu X., Zhao X., Chen L.-J., Ma P., Liu T., Yan X.-P. (2023). Mesoporous polyacrylic acid/calcium phosphate coated persistent luminescence nanoparticles for improved afterglow bioimaging and chemotherapy of bacterial infection. Biomater. Sci..

[195] Wang R., Shi J., Song L., Zheng S., Liu X., Hong M., Zhang Y. (2021). Sustained Antitumor Immunity Based on Persistent Luminescence Nanoparticles for Cancer Immunotherapy. Adv. Funct. Mater..

[196] Shu G., Zhu W., Jiang Y., Li X., Pan J., Zhang X., Zhang X., Sun S.-K. (2021). Persistent Luminescence Immune Hydrogel for Photodynamic-Immunotherapy of Tumors In Vivo. Adv. Funct. Mater..

[197] Li Y., Teng X., Wang Y., Yang C., Yan X., Li J. (2021). Neutrophil Delivered Hollow Titania Covered Persistent Luminescent Nanosensitizer for Ultrosound Augmented Chemo/Immuno Glioblastoma Therapy. Adv. Sci..

